# Spatiotemporal Transcriptomics Characterizes Immune Microenvironment During Mouse Liver Aging

**DOI:** 10.1111/acel.70482

**Published:** 2026-04-20

**Authors:** Jiahua Lu, Yuqian Wang, Wenxue Zhao, Zihao Zhao, Zhaoya Gao, Jin Gu, Cheng Li, Jie Cheng

**Affiliations:** ^1^ Department of Pathology, School of Basic Medicine, Tongji Medical College and State Key Laboratory for Diagnosis and Treatment of Severe Zoonotic Infectious Diseases Huazhong University of Science and Technology Wuhan Hubei China; ^2^ Institute of Pathology, Tongji Hospital Huazhong University of Science and Technology Wuhan Hubei China; ^3^ Center for Precision Medicine Multi‐Omics Research, Institute of Advanced Clinical Medicine Peking University Beijing China; ^4^ School of Life Sciences, Center for Bioinformatics, Center for Statistical Science Peking University Beijing China; ^5^ Department of Gastrointestinal Surgery Peking University Shougang Hospital Beijing China; ^6^ Center for Precision Diagnosis and Treatment of Colorectal Cancer and Inflammatory Diseases Peking University Health Science Center Beijing China; ^7^ Peking University International Cancer Institute Peking University Beijing China; ^8^ Peking‐Tsinghua Center for Life Sciences Peking University Beijing China; ^9^ Key Laboratory of Carcinogenesis and Translational Research (Ministry of Education/Beijing), Department of Gastrointestinal Surgery Peking University Cancer Hospital & Institute Beijing China

**Keywords:** aging, liver, single‐cell/nuclei transcriptome, spatial transcriptome, T cell exhaustion

## Abstract

The liver is a major metabolic organ, responsible for synthesizing and breaking down diverse metabolites. Recently, the liver's immunological functions have gradually been unveiled: combating pathogens and maintaining tissue homeostasis. Age‐related functional alterations in these immune cells emerge as potential drivers of hepatic dysfunction and age‐associated pathologies. However, systematic investigations into spatiotemporal immune cell dynamics during liver aging remain limited. To address this gap, we analyzed young and old mouse livers using single‐cell/nuclei and spatial transcriptomics, revealing T cells as the immune cell population with the most pronounced transcriptomic alterations, marked by enrichment of exhausted CD8^+^ T cells in aged livers. Spatial mapping showed exhausted CD8^+^ T cells accumulating in portal vein (PV) zone, co‐localizing with periportal hepatocytes (PP hepatocytes). Up‐regulation of LPIN1 in PP hepatocyte promoted T cell exhaustion. CD8^+^ T cell exhaustion was tightly associated with disease progression. Therefore, our findings suggest that targeting LPIN1 may alleviate T cell exhaustion, offering potential therapeutic strategies for age‐related liver diseases.

## Graphical abstract

T cells are the most dynamically altered immune population in the aged liver. Accumulation of CD8^+^ Tex in aged livers. Spatial enrichment of CD8^+^ Tex in the portal vein zone. PP hepatocytes promote T cell exhaustion via *Lpin1*. T cell exhaustion correlates with liver disease progression.

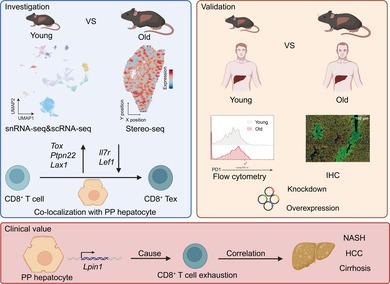

## 1 Introduction

The liver, as the largest organ in the human body, plays a crucial role in maintaining metabolic homeostasis (Ishibashi et al. 2009). Its unique spatial architecture results in distinct functional properties among hepatocytes in different zones (Ben‐Moshe and Itzkovitz 2019). Beyond its metabolic functions, the liver is increasingly recognized for its immunological roles, housing various immune cells such as Kupffer cells, dendritic cells (DCs), and T cells (Robinson et al. 2016). These immune cells are pivotal in antigen presentation, modulation of inflammatory responses, and clearance of pathogens from gut and damaged cells, underscoring the liver's dual role as both a metabolic hub and an immunological sentinel (Robinson et al. 2016).

Despite the liver's critical functions, aging leads to a decline in its performance, increasing the risk of liver‐related diseases such as hepatitis, fibrosis, cirrhosis, and hepatocellular carcinoma (HCC). Current research into liver aging has predominantly focused on non‐immune cells, such as hepatocytes, endothelial cells, and hepatic stellate cells, revealing their functional alterations and contributions to age‐related pathologies (Sanfeliu‐Redondo et al. 2024). However, the dynamic changes and functional roles of immune cells within the aged liver remain poorly understood. This gap in knowledge hinders a comprehensive understanding of the mechanisms underlying age‐associated liver diseases and the development of targeted therapies.

Recently, the roles of immune cells in liver aging have begun to be uncovered. For instance, Mogilenko et al. identified the accumulation of GZMK^+^ CD8^+^ T cells in aged livers and suggested their potential role in promoting cellular senescence (Mogilenko et al. 2021). Similarly, Liu et al. reported increased proportions of Cxcl2^+^ macrophages in aged livers, which recruit neutrophils and contribute to age‐related liver injury (Liu et al. 2024). However, both studies lack spatial resolution, failing to capture the microenvironmental interactions of immune cells within the liver's unique zonal architecture. To address these limitations, our study employed single‐cell/nuclei and spatial transcriptomics to comprehensively map the dynamic changes and spatial heterogeneity of immune cells in young and aged mouse livers. T cells are identified as the most transcriptionally dynamic immune cell type, characterized by exhaustion markers and associated with disease progression. Furthermore, we explored the intra‐ and extracellular factors contributing to exhaustion and discovered that PP hepatocytes may promote the exhaustion of neighboring T cells through the upregulation of LPIN1. The findings offer new perspectives for understanding age‐related liver pathologies and developing therapeutic strategies.

## 2 Result

### 2.1 Single Cell Transcriptional Analysis Revealed the Landscape of Immune Cells During Liver Aging and T Cells Exhibit the Most Pronounced Changes

**FIGURE 1:**
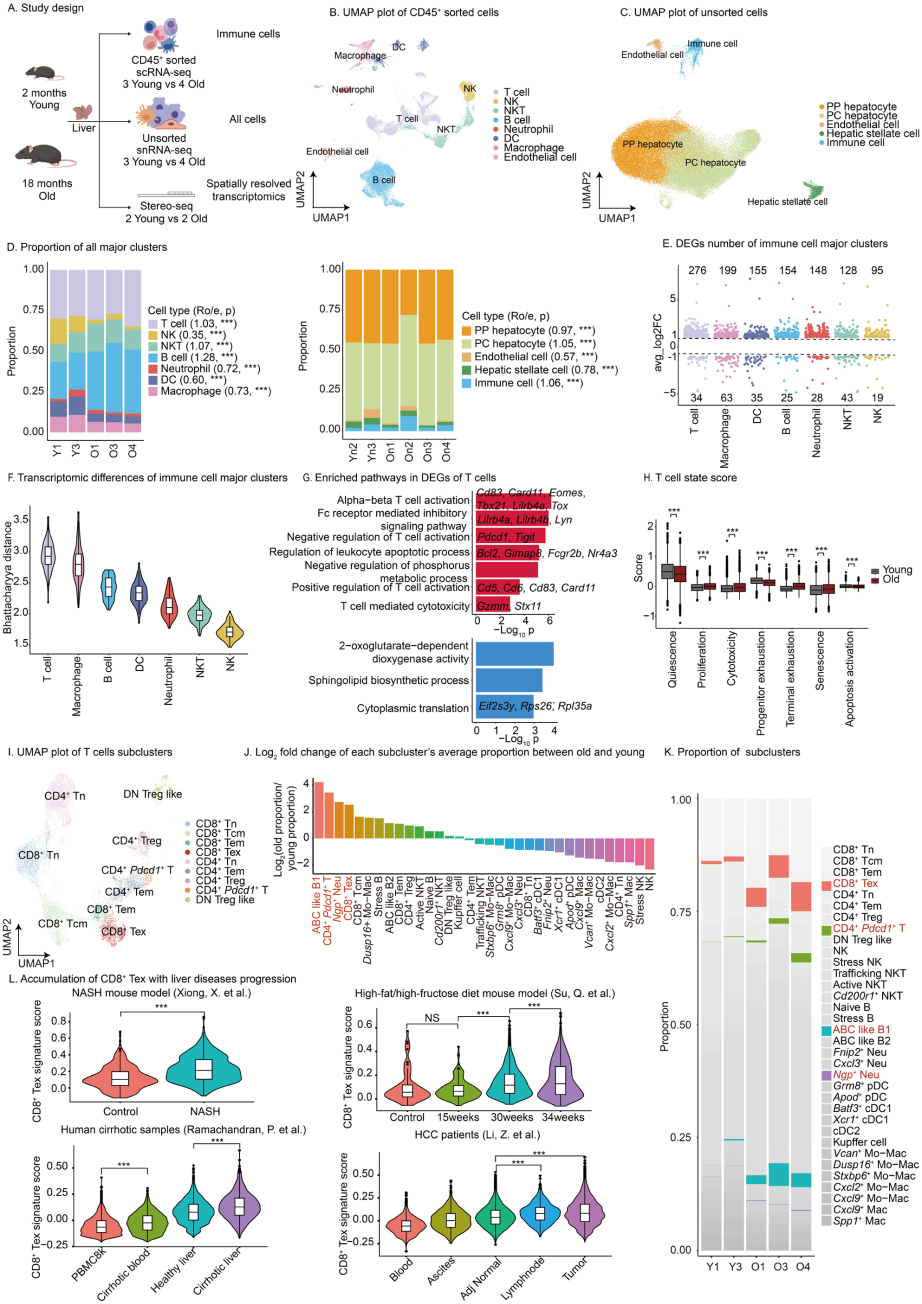
Single‐cell RNA sequencing (scRNA‐seq) and single‐nuclei RNA sequencing (snRNA‐seq) revealed changes of T cell during liver aging and its role in diseases progression. (A) Experimental design workflow. Livers from young group (2 months) and old group (18 months) were used for multi‐omics sequencing. CD45^+^ sorted scRNA‐seq was applied on 3 young livers and 4 old livers to capture immune cells. Unsorted snRNA‐seq was utilized on 3 young livers and 4 old livers to capture all cells. Stereo‐seq was employed on 2 young livers and 2 old livers to capture spatial information of aging microenvironment. Figure was created with BioGDP.com. (B) Uniform Manifold Approximation and Projection (UMAP) plot of CD45^+^ sorted cells. Clusters are colored by major cell types. (C) UMAP plot of unsorted cells. Clusters are colored by major cell types. (D) Major cell type composition. Colors are consistent with those in the plot B and C. Ro/e, observed number in old group versus expected number in old group. Statistical significance is tested using the chi‐square test. ***p < 0.001. (E) Differentially expressed genes (DEGs) number of immune cells. Major cell types are sorted based on the total number of DEGs. The numbers above the dot plot represent the number of upregulated genes, while those below represent the number of downregulated genes. Colors are consistent with those in the plot B. (F) Bhattacharyya distance between old and young groups. Major cell types are sorted based on the median of Bhattacharyya distance. Colors are consistent with those in the plot B. (G) T cell DEGs enrichment analysis. Red represents upregulated pathways, blue represents downregulated pathways. Representative genes within the pathways are displayed. (H) Score of T cell state pathway. Red represents old group, gray represents young group. Statistical significance is tested using the two‐tailed unpaired *t*‐test with heteroscedasticity. ***p < 0.001. (I) UMAP plot of T cells. Clusters are colored by subclusters. (J) Log2 fold change of cell proportion. Subclusters are sorted based on the log2 fold change of old group mean proportion versus young group mean proportion. Colors are assigned in order. The four most divergent subclusters are highlighted in red. (K) Subclusters proportion. The four most divergent subclusters are highlighted in red. (B–K) scRNA‐seq and snRNA‐seq data were generated in house. (L) CD8^+^ Tex signature score in CD8+ T cells. NASH, non‐alcoholic steatohepatitis. 15/30/34 weeks, duration of high‐fat, high‐fructose diet. scRNA‐seq data were collected from public databases. Statistical significance is tested using the two‐tailed unpaired *t*‐test with heteroscedasticity. NS *p* ≥ 0.05, ***p < 0.001.

To characterize the landscape of immune cells during liver aging, single‐cell RNA sequencing (scRNA‐seq) was employed. Given that immune cells are low abundance in livers, CD45^+^ sorted cells were captured for scRNA‐seq (Figure 1A). To investigate the interaction between non‐immune cells and immune cells, and considering hepatocytes tend to die during experiments, single‐nuclei RNA sequencing (snRNA‐seq) was utilized (Figure 1A). High quality single cells and single nuclei were obtained after stringent quality control and low‐quality samples (Y2, O2, and Yn1) were excluded from further processing (Figure S1A,B). To identify major cell types, unsupervised clustering was used to get 8 cell types from scRNA‐seq data and 5 cell types from snRNA‐seq data (Figure 1B,C, Figure S1C–G). Collectively, we constructed a whole cell types atlas of young and old mouse livers.

Then we explored the composition and transcriptome changes during liver aging. Cell enrichment analysis (Chu et al. 2024) reveals all cell types have significant compositional shifts. The old group exhibits significant reductions in proportions of NK cells, neutrophils, DCs, macrophages, PP hepatocytes, endothelial cells, and hepatic stellate cells, while showing increased proportions in T cells, NKT cells, B cells, pericentral hepatocytes (PC hepatocytes), and total immune cells (Figure 1D). This cellular redistribution pattern aligns with previously reported immune cell proportion dynamics in liver aging research (Liu et al. 2024; Lin et al. 2024; Yang et al. 2023). Batch effects are mixed with group differences in snRNA‐seq data; therefore, we only analyzed immune cell transcriptomic changes. Notably, T cells show the most pronounced changes among immune cells, with the highest bhattacharyya distance and the most differentially expressed genes (DEGs) (Figure 1E,F). Together, these data reveal that T cells increase in population and change greatly in their transcriptome during liver aging.

Next, we investigated the functional changes of T cells. DEGs enrichment analysis reveals that both positive and negative regulation of T cell activation, as well as the cytotoxic pathway, are significantly upregulated in aged livers (Figure 1G). *Cd5* and *Cd83* are activation markers of T cells (Cramer et al. 2000; Biancone et al. 1996); *Cd6* enhances T cell receptor (TCR) signaling amplification (Hassan et al. 2006); *Card11* mediates TCR pathway transduction (Hwang et al. 2020); *Eomes* and *Tbx21* associate with cytotoxic and memory functions in activated T cells (Intlekofer et al. 2005). *Tox*, *Pdcd1*, and *Tigit* serve as exhaustion markers in T cells (Khan et al. 2019; Wherry and Kurachi 2015); *Lilrb4a/b* suppress T cell functionality (Deng et al. 2018); *Lyn* mediates immunosuppressive signaling (Mkaddem et al. 2017). *Gzmm* encodes granzyme M, and *Stx11* mediates granzyme release (Chiang et al. 2013). Besides, in aged livers, the apoptosis regulation pathway is upregulated, with *Bcl2* and *Gimap8* inhibiting apoptosis (Jourdan et al. 2009; Ho and Tsai 2017) and *Fcgr2b* and *Nr4a3* promoting it (Morris et al. 2020; Fedorova et al. 2019) (Figure 1G). Negative regulation of the phosphorus metabolic pathway is upregulated in aged livers; cytoplasmic translation initiation factor (*Eif2s3y*) and ribosomal proteins (*Rps26, Rpl35a*) are downregulated (Figure 1G). This metabolic shift coupled with diminished translational capacity suggests T cell senescence. To further delineate global T cell states, pathway scoring demonstrates that during liver aging, T cells exit quiescence with enhanced proliferation/cytotoxicity, yet display terminal exhaustion/senescence differentiation alongside inhibited apoptosis, consistent with senescence progression (Figure 1H). These results indicate that T cells exhibit functional changes, showing exhaustion and senescence features in aged livers.

To dissect T cell heterogeneity as well as other immune cells, we performed sub‐clustering of major cell types (Figure 1I, Figures S2 and S3). Proportion fold change analysis shows that age‐associated B cell like B cell type 1 (ABC like B1), *Pdcd1* positive CD4^+^ T cell (CD4^+^ *Pdcd1* ^+^ T), *Npg* positive neutrophil (*Npg* ^+^ Neu), and exhausted CD8^+^ T cell (CD8^+^ Tex) are the four cell types exhibiting the most proportional changes (Figure 1J, Figure S4B–D). Furthermore, cell enrichment analysis (Chu et al. 2024) confirmed the significance (Figure S4A). Among the four cell types, CD8^+^ Tex exhibits the highest proportion in the old group suggesting its important role (Figure 1K). To further validate the accumulation of CD8^+^ Tex, we collected public scRNA‐seq datasets (Mogilenko et al. 2021; Almanzar 2020; Cai et al. 2023; Nikopoulou et al. 2023) and obtained the same conclusion (Figure S4E–H). Altogether, CD8^+^ Tex accumulates in aged livers which is consistent with the functional changes of T cells.

To elucidate the relationship between CD8^+^ Tex accumulation and liver disease progression, we analyzed CD8^+^ T cell exhaustion state in multiple disease conditions. In non‐alcoholic steatohepatitis (NASH) mouse models (Xiong et al. 2019), CD8^+^ T cells in livers with NASH exhibit higher exhaustion score (Figure 1L). High‐fat/high‐fructose diet mice (Su, Kim, et al. 2021) show diet‐duration‐dependent NASH aggravation with elevated CD8^+^ Tex signature score, implicating pathogenic role of CD8^+^ Tex (Figure 1L). Human cirrhotic livers (Ramachandran et al. 2019) exhibit CD8^+^ T cells resembling CD8^+^ Tex in aged livers, with concurrent elevation of CD8^+^ Tex signature score in peripheral blood versus control, indicating systemic CD8^+^ T cell dysregulation (Figure 1L). In HCC patients (Li et al. 2024), CD8^+^ T cells in lymph nodes and tumor tissues show higher CD8^+^ Tex signature scores compared to non‐tumor tissues (Figure 1L). Dudek et al. reported CXCR6^+^ CD8^+^ auto‐aggressive Tex accumulation in NASH murine/human livers (Dudek et al. 2021), while CD8^+^ Tex lacks comparable gene signatures (Figure S4I,J). Collectively, CD8^+^ T cells across liver pathologies show increased transcriptional similarity to aged CD8^+^ Tex, in correlation level, implicating their pathogenic role.

### 2.2 T Cells in Aged Livers Exhibit Unique Exhaustion Pathway With Heightened Exhaustion Level

**FIGURE 2:**
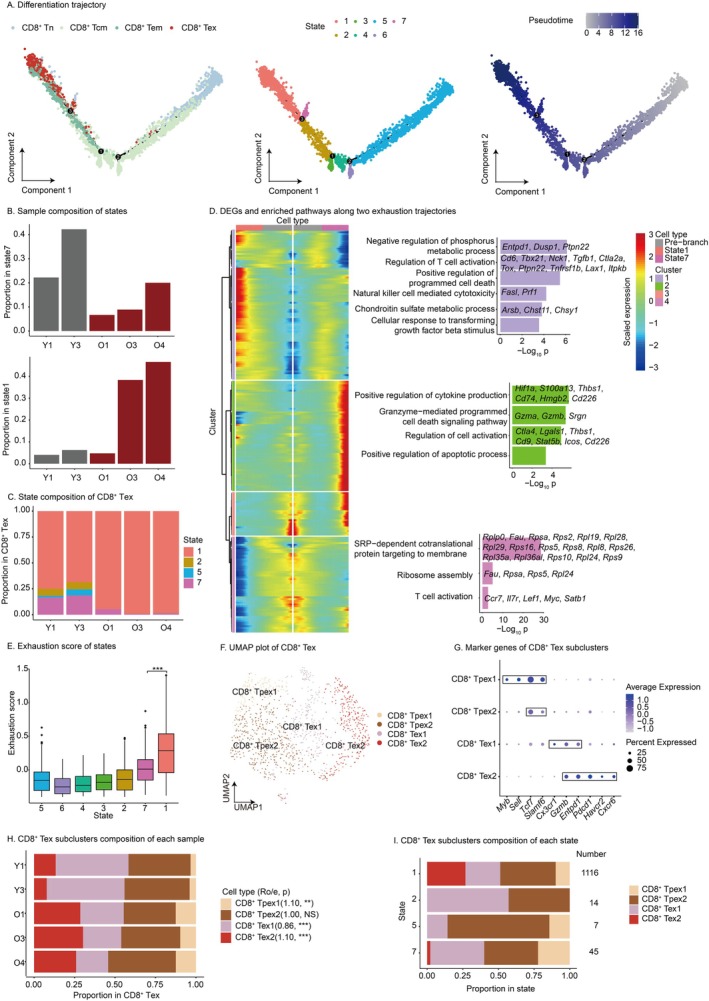
Intrinsic factors of CD8^+^ T cell exhaustion. (A) Differentiation trajectory of CD8^+^ T cells. Left, subclusters distribution in trajectory; colors are consistent with those in the UMAP plot. Middle, state distribution in trajectory; colors represent the states. Right, pseudotime distribution in trajectory; colors represent the pseudotime. (B) Sample composition of state. Upper, state7. Bottom, state1. Red represents the old group and gray represents the young group. (C) State composition of CD8^+^ Tex. Colors are consistent with those in the plot A. (D) DEGs and enriched pathways in two exhaustion trajectories. Left, DEGs along two exhaustion trajectories. Colors represent scaled average expression. DEGs are grouped into 4 clusters: cluster1 represents upregulated genes in state1; cluster2 represents upregulated genes in state7; cluster3 represents low grade downregulated genes in state1; cluster4 represents downregulated genes in state1. Right, enriched pathways. Colors are consistent with clusters. Representative genes within the pathways are displayed. (E) Exhaustion score of each state. Colors are consistent with those in the plot A. Statistical significance is tested using the two‐tailed unpaired *t*‐test with heteroscedasticity. ***p < 0.001. (F) UMAP plot of CD8^+^ Tex. Clusters are colored by subclusters. (G) Marker genes of CD8^+^ Tex subclusters. Colors represent scaled average expression. Dot size represents the percentage of cells that express the gene. Marker genes of the same subcluster are highlighted with a box. (H) CD8^+^ Tex subclusters composition of each sample. Colors are consistent with those in the plot G. Ro/e, observed number in old group versus expected number in old group. Statistical significance is tested using the chi‐squared test. NS *p* ≥ 0.05, **p < 0.01, ***p < 0.001. (I) CD8^+^ Tex subclusters composition of each state. Colors are consistent with those in the plot G. Numbers to the right of the bar represent the cell numbers. (A–I) scRNA‐seq data were generated in house.

To investigate intrinsic factors promoting CD8^+^ T cell exhaustion, we conducted a CD8^+^ T cell differentiation trajectory. The trajectory delineates CD8^+^ T cell transition from naïve to memory and ultimately exhausted state (Figure 2A). Notably, there are two exhaustion pathways, one involving state7 and the other involving state1 (Figure 2A). Analysis shows that CD8^+^ Tex in state7 mostly come from the young group, while CD8^+^ Tex in state1 mostly come from the old group (Figure 2B). Further analysis of CD8^+^ Tex composition in each sample shows that the young group's CD8^+^ Tex partly comes from state7, whereas the old group's CD8^+^ Tex almost entirely comes from state1 (Figure 2C). This suggests that the differentiation pathway leading to state1 is preferentially active in the old group.

Therefore, to elucidate CD8^+^ Tex accumulation in aged livers, we analyzed the DEGs of pathway leading to state1. Enrichment analysis reveals state1 upregulates T cell suppression genes (Figure 2D): *Tox* drives exhaustion (Khan et al. 2019); *Ptpn22* inhibits ZAP70/LCK phosphorylation (Tizaoui et al. 2021); *Tnfrsf1b* suppresses IFNγ in exhausted T cells (Gao et al. 2023); *Lax1* impairs TCR signaling (Zhu et al. 2005); *Itpkb* deletion enhances T cell activation/memory phenotypes and cytotoxicity (Pouillon et al. 2013). Additionally, state1 downregulates genes maintaining stemness and memory (Figure 2D): *Ccr7* mediates lymph node homing (Förster et al. 2008); *Il7r* maintains memory T cells (Barata et al. 2019); *Lef1* sustains stem‐like cytotoxic T cells and is essential for enhanced immune checkpoint blockade response (Zhao et al. 2022); *Myc* drives proliferation‐associated metabolism in activated T cells (Rathmell 2011); *Satb1* suppresses PD1 upregulation during activation (Stephen et al. 2017). Besides, state1 upregulated genes are enriched in metabolic pathways, including negative regulation of phosphorus metabolic process and chondroitin sulfate metabolism, suggesting metabolic reprogramming. Cytotoxic pathway based on perforin and death ligands is also enriched by upregulated genes. Downregulated genes are enriched in co‐translation and ribosome assembly pathways, suggesting reduced translation capacity and cellular activity during liver aging (Figure 2D). State7 drives exhaustion through upregulation of *Ctla4*, *Lgals1* and *Thbs1* (Walker and Sansom 2011; Rubinstein et al. 2004; Omatsu et al. 2023), while gaining enhanced cytokine production and granzyme‐mediated cytotoxicity (Figure 2D). Although DEGs of state1 are enriched in TGFβ response pathway, and both DEGs of state1 and state7 are enriched in apoptosis pathway, no significant differences are observed in the overall pathway scores (Figure S4K). Altogether, these analyses reveal that in the process of liver aging, T cells undergo aging‐specific exhaustion by upregulating specific exhaustion‐related genes and downregulating memory/stemness‐related genes.

Next, we explored the exhaustion level of CD8^+^ Tex. Scoring with exhaustion genes (*Pdcd1*, *Lag3*, *Tigit*, *Tox*, *Havcr2*, *Cxcl13*) reveals significantly higher scores in state1 compared to state7 (Figure 2E), suggesting greater exhaustion level in the old group‐enriched state1. To further validate the phenomena, we employed sub‐clustering on CD8^+^ Tex according to previous researches (Zhou et al. 2023; Beltra et al. 2020) (Figure 2F,G, Figure S4L). Four distinct exhaustion states were identified: precursor exhausted type 1 (CD8^+^ Tpex1), precursor exhausted type 2 (CD8^+^ Tpex2), terminally exhausted type 1 (CD8^+^ Tex1), and terminally exhausted type 2 (CD8^+^ Tex2), with progressively increasing exhaustion levels. Cell enrichment analysis reveals increased CD8^+^ Tpex1 and CD8^+^ Tex2 populations in the old group, whereas CD8^+^ Tex1 is enriched in the young group, indicating that the old group exhibits not only expanded exhausted T cell sources but also heightened exhaustion level. Further analysis of subset proportions across states demonstrated that state1 (enriched in the old group) contains more CD8^+^ Tex2, concordant with its higher exhaustion gene score (Figure 2H,I).

### 2.3 CD8 ^+^ Tex Is Specifically Enriched in the PV Zone of the Aged Liver

**FIGURE 3:**
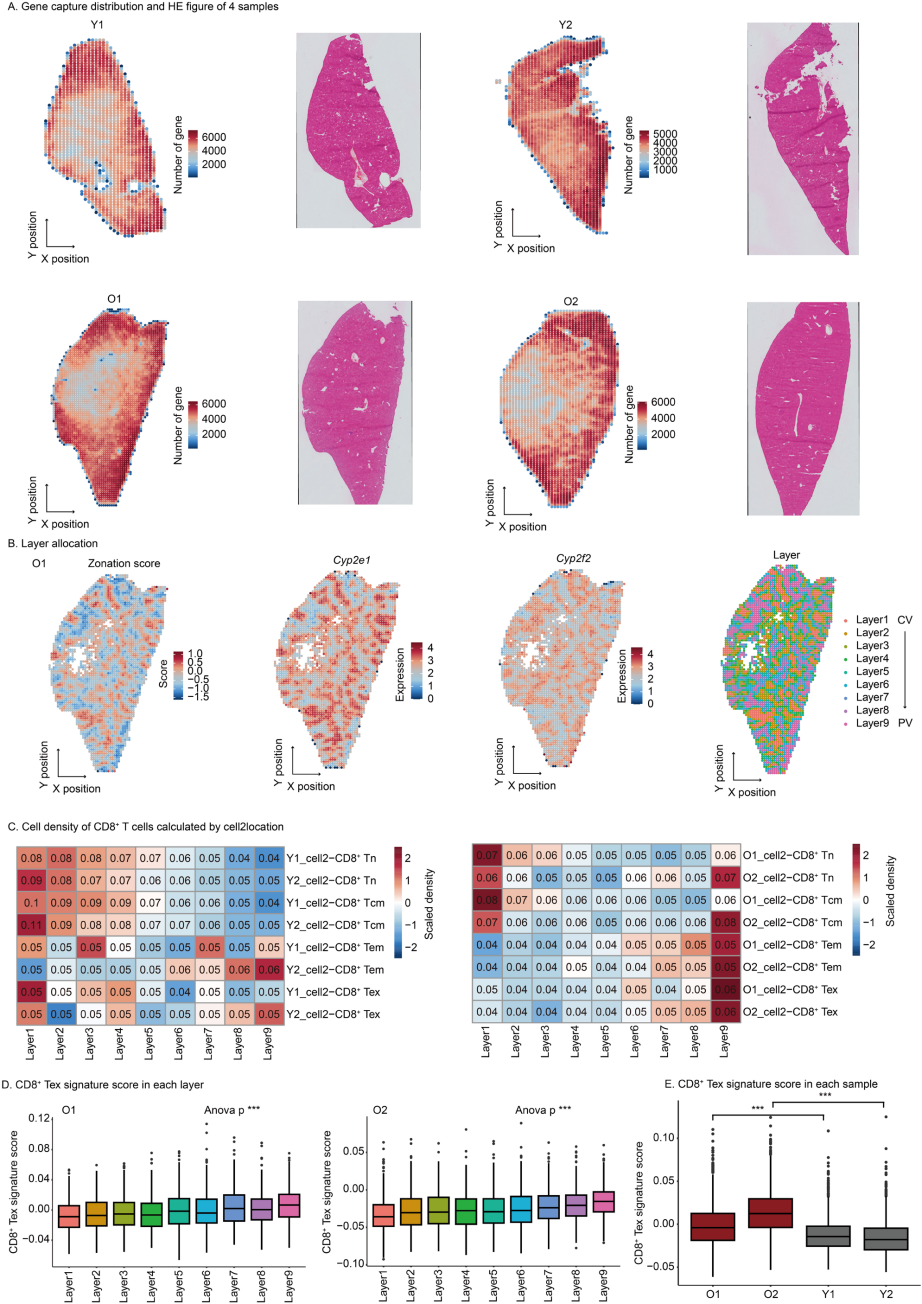
CD8^+^ Tex preferred to locate in portal vein (PV) zone. (A) Number of captured genes and HE plot. A spot is composed of 200 bins. Colors represent the number of genes. (B) Layer allocation. The first three subplots represent the spatial distribution of region score, central vein (CV) zone marker gene *Cyp2e1*, and PV zone marker gene *Cyp2f2*, respectively. The color intensity indicates higher scores or expression levels in red and lower values in blue. The rightmost subplot shows the layer classification, with layers 1–9 representing the transition from CV zone to PV zone. (C) Cell density distribution of CD8^+^ T cells. The color gradient represents scaled density values, with red indicating higher values and blue indicating lower values. The numbers within each box show the raw (unscaled) density. (D) CD8^+^ Tex signature score in each layer. Statistical significance is tested using the anova. ***p < 0.001. (E) CD8^+^ Tex signature score in each sample. Statistical significance is tested using the two‐tailed unpaired *t*‐test with heteroscedasticity. ***p < 0.001. (A–E) Stereo‐seq data were generated in house.

To dissect the microenvironment of CD8^+^ Tex, we performed Stereo‐seq and spatial analyses (Figures 1A and 3A). Bin200 was chosen as a spatial spot and underwent quality control (Figure 3A, Figure S5A). The liver exhibits unique spatial heterogeneity (Ben‐Moshe and Itzkovitz 2019), therefore, following a previous study (Wu et al. 2024), spots were divided into 9 layers to investigate the spatial distribution of CD8^+^ Tex (Figure 3B, Figure S5B). Given that each spot contains multiple cells, we used the scRNA‐seq and snRNA‐seq data generated in this study to annotate the Stereo‐seq data. The hepatocyte density distribution annotated by cell2location (Kleshchevnikov et al. 2022) aligns with theory, demonstrating the reliability of the annotation results (Figure S6A). The density distribution of CD8^+^ T cell subclusters was observed, revealing that CD8^+^ Tex is specifically enriched in layer 6–9 (PV zone) of the old group (Figure 3C, Figure S6E). Stereoscope (Andersson et al. 2020) annotation corroborated the same conclusion (Figure S6B,C,E). To further validate the spatial distribution of CD8^+^ Tex, the signature gene scoring method was employed for analysis. The distribution of scores for endothelial cells aligns with expectations, indicating the reliability of this method (Figure S6D). The CD8^+^ Tex shows a progressive increase in scores from the CV zone to PV zone in the old group, while other subclusters lack this spatial trend (Figure 3D, Figure S7). Additionally, CD8^+^ Tex scores are significantly higher in the old group compared to the young group, corroborating findings from scRNA‐seq data analysis (Figure 3E). Collectively, CD8^+^ Tex predominantly accumulates in the PV zone of the aged liver.

### 2.4 CD8 ^+^ Tex Co‐Localizes With PP Hepatocyte in the Aged Liver

**FIGURE 4:**
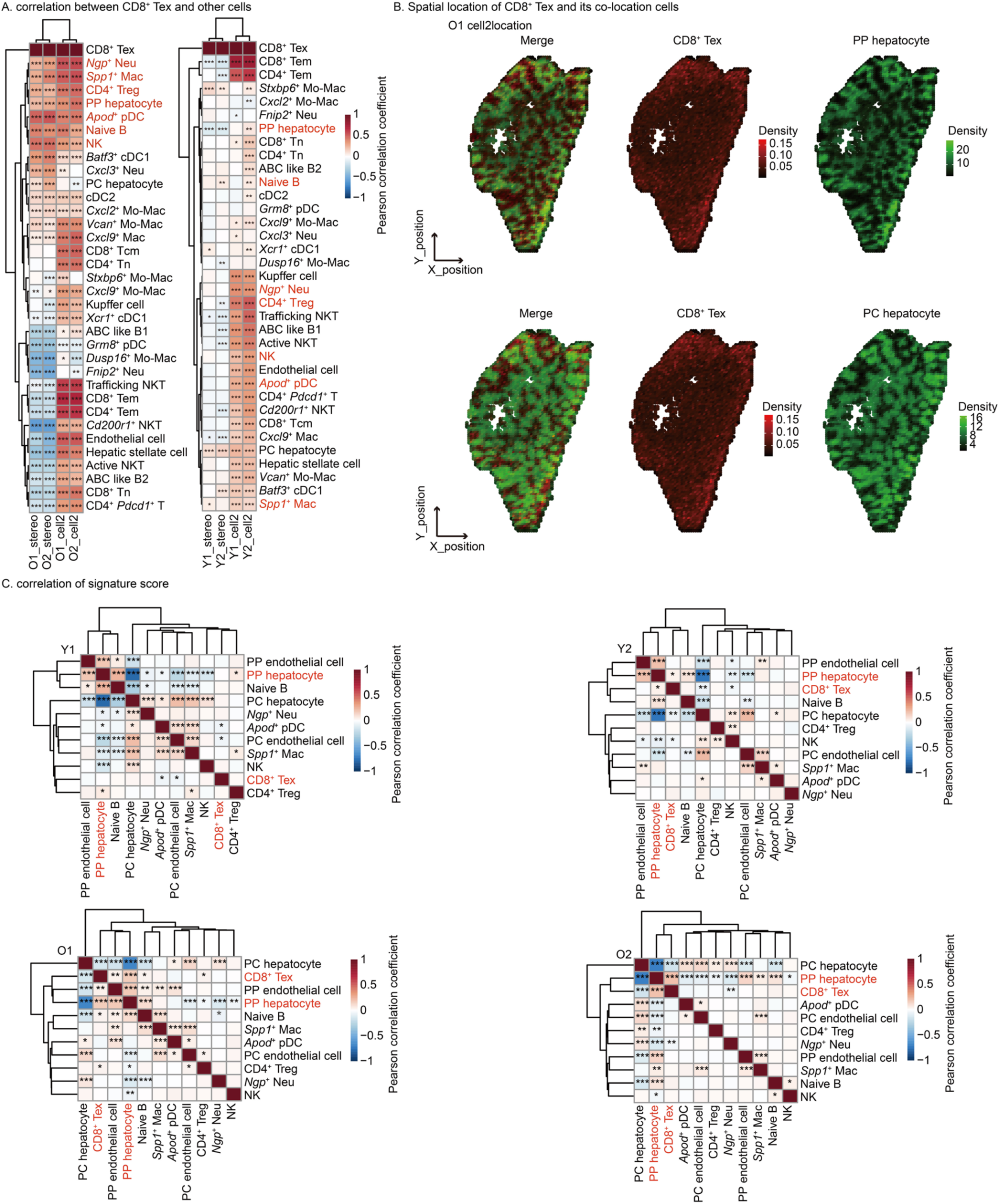
CD8^+^ Tex and periportal hepatocyte (PP hepatocyte) co‐location. (A) Density/Proportion correlation between CD8^+^ Tex and other subclusters. Potential CD8^+^ Tex co‐localizing subclusters are highlighted in red. Colors represent pearson correlation coefficient. *p < 0.05, **p < 0.01, ***p < 0.001. (B) Density distribution. Color represents density. In monochrome mode, brighter shades indicate higher density, while darker shades indicate lower density. “Merge” denotes the blending of colors corresponding to the densities of two subclusters. (C) Correlation of signature gene scores. Potential CD8^+^ Tex co‐localizing subclusters are highlighted in red. Colors represent pearson correlation coefficient. *p < 0.05, **p < 0.01, ***p < 0.001. (A–C) Stereo‐seq data were generated in house.

In addition to delineating the spatial distribution of CD8^+^ Tex, we further investigated their co‐localized cellular partners by conducting a cellular co‐localization analysis. Analysis of cell density and proportion correlations reveals that CD8^+^ Tex exhibits co‐localization with diverse cell types in the old group, including *Ngp* ^+^ Neu, *Spp1* ^+^ Mac, CD4^+^ Treg, PP hepatocyte, *Apod* ^+^ pDC, Naïve B and NK (Figure 4A). Signature gene score‐based correlation analysis further delineated co‐localized cell partners (Figure S8). A significant positive correlation is observed between hepatocytes and endothelial cells signature scores in the same anatomical zone, validating the method's capability to detect spatially co‐localized cell types (Figure 4C). In the end, among all potential co‐localized cell types with CD8^+^ Tex, only PP hepatocytes exhibited a significant positive correlation in their scoring profiles (Figure 4C). Furthermore, we validated the colocalization of CD8^+^ Tex with aged PP hepatocytes using a colocalization score—a non‐correlation‐based method (Figure S9B). In summary, the spatial overlap of CD8^+^ Tex and PP hepatocyte in the old group indicates that PV‐specific Tex accumulation may be driven by PP hepatocytes (Figure 4B, Figure S9A).

### 2.5 PP Hepatocytes of Aged Livers May Induce T Cell Exhaustion Through Metabolic Pathways

**FIGURE 5:**
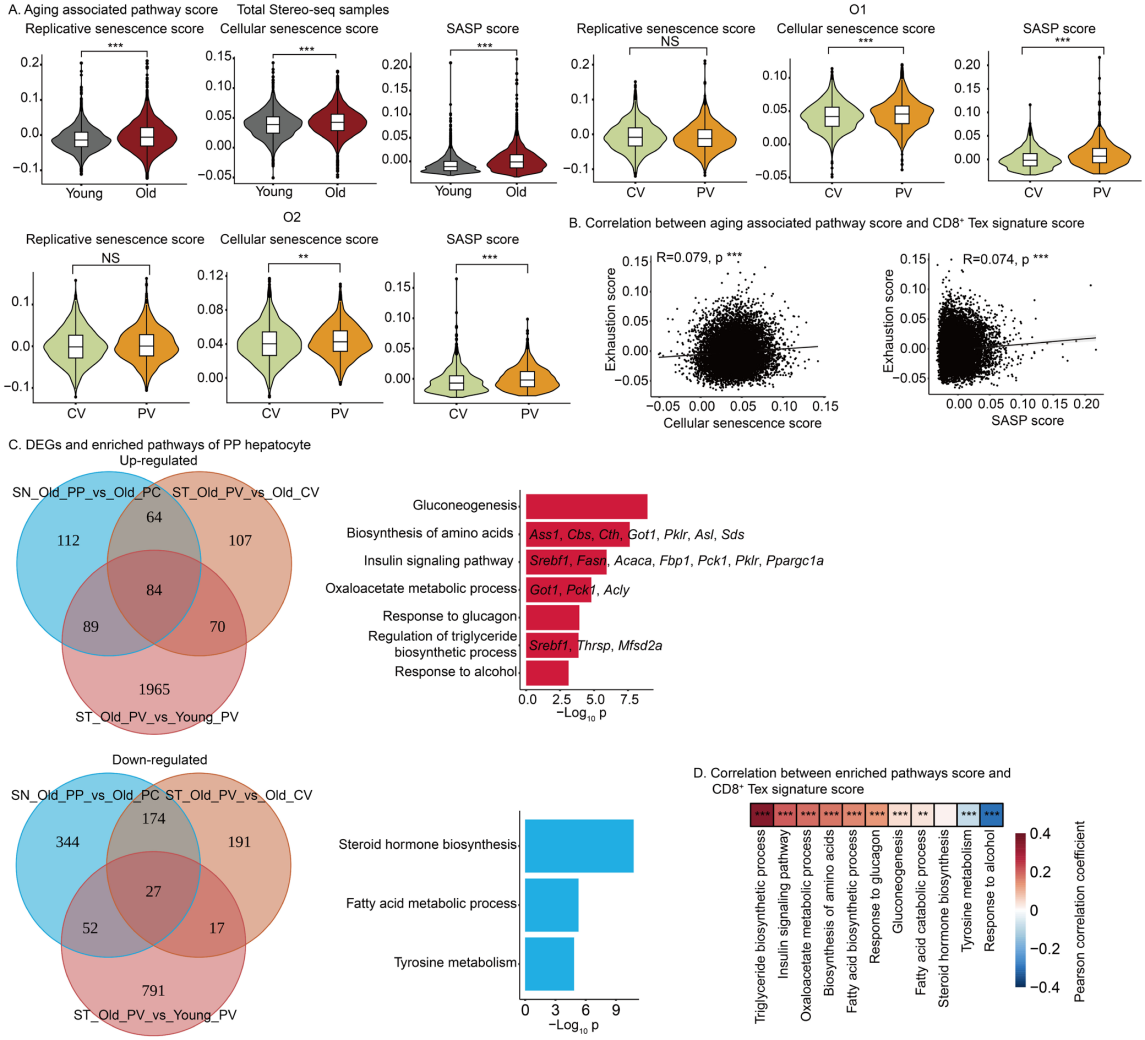
Metabolites produced by PP hepatocyte may induce T cell exhaustion. (A) Aging associated pathway scores across groups and zones. SASP, senescence‐associated secretory phenotype. Statistical significance is tested using the two‐tailed unpaired *t*‐test with heteroscedasticity. NS *p* ≥ 0.05, **p < 0.01, ***p < 0.001. (B) Correlation between aging associated pathway score and CD8^+^ Tex signature score. The shaded area represents the 95% confidence interval. ***p < 0.001. (C) DEGs number and enriched pathways of PP hepatocyte. Left, the upper Venn diagram represents the intersection of upregulated genes, while the lower one corresponds to downregulated genes. Right, enriched pathways: red represents upregulated pathways, blue represents downregulated pathways. Representative genes within the pathways are displayed. (D) Correlation between metabolic pathway score and CD8^+^ Tex signature score. Colors represent pearson correlation coefficient. **p < 0.01, ***p < 0.001. (A–D) Stereo‐seq and snRNA‐seq data were generated in house.

To explore the relationship between PP hepatocytes and T cell exhaustion, we analyzed transcription characteristics of PP hepatocytes in the old group. Given the age‐specific accumulation of CD8^+^ Tex, it is critical to investigate whether aging‐related pathways are mechanistically linked to T cell exhaustion. Consistent with expectations, three senescence‐related pathways—replicative senescence, cellular senescence, and senescence‐associated secretory phenotype (SASP)—exhibit significantly higher scores in the old group compared to the young group (Figure 5A). The cellular senescence and SASP pathways show significantly higher scores in the PV zone compared to the CV zone, a pattern consistently observed in both the young and old groups (Figure 5A, Figure S10A). While CV and PV zones show distinct senescence levels, cellular senescence and SASP activity do not correlate with CD8^+^ Tex signature score, indicating that local senescence has little effect on T cell dysfunction (Figure 5B).

Next, we analyzed DEGs of PP hepatocytes from the old group. Gene enrichment analysis was performed on the intersection of three gene sets: PP hepatocytes of the old group versus PC hepatocytes of the old group in snRNA‐seq data; PV zone of the old group versus CV zone of the old group in Stereo‐seq data; PV zone of the old group versus PV zone of the young group in Stereo‐seq data (Figure 5C). DEGs are significantly enriched in multiple metabolism‐related pathways, with upregulated genes specifically showing enrichment in amino acid biosynthesis, regulation of triglyceride biosynthesis, oxaloacetate metabolism, and insulin signaling pathways (Figure 5C). The activity scores of these four pathways are consistently higher in the PV zone or PP hepatocytes across all three groups (Figure S10B). Notably, the activity scores of these four pathways exhibit strong positive spatial correlations with CD8^+^ Tex signature score, suggesting that these pathways may drive T cell exhaustion in the aged liver microenvironment (Figure 5D).

### 2.6 PP Hepatocytes in Aged Livers Promote T Cell Exhaustion via Elevated LPIN1 Expression

**FIGURE 6:**
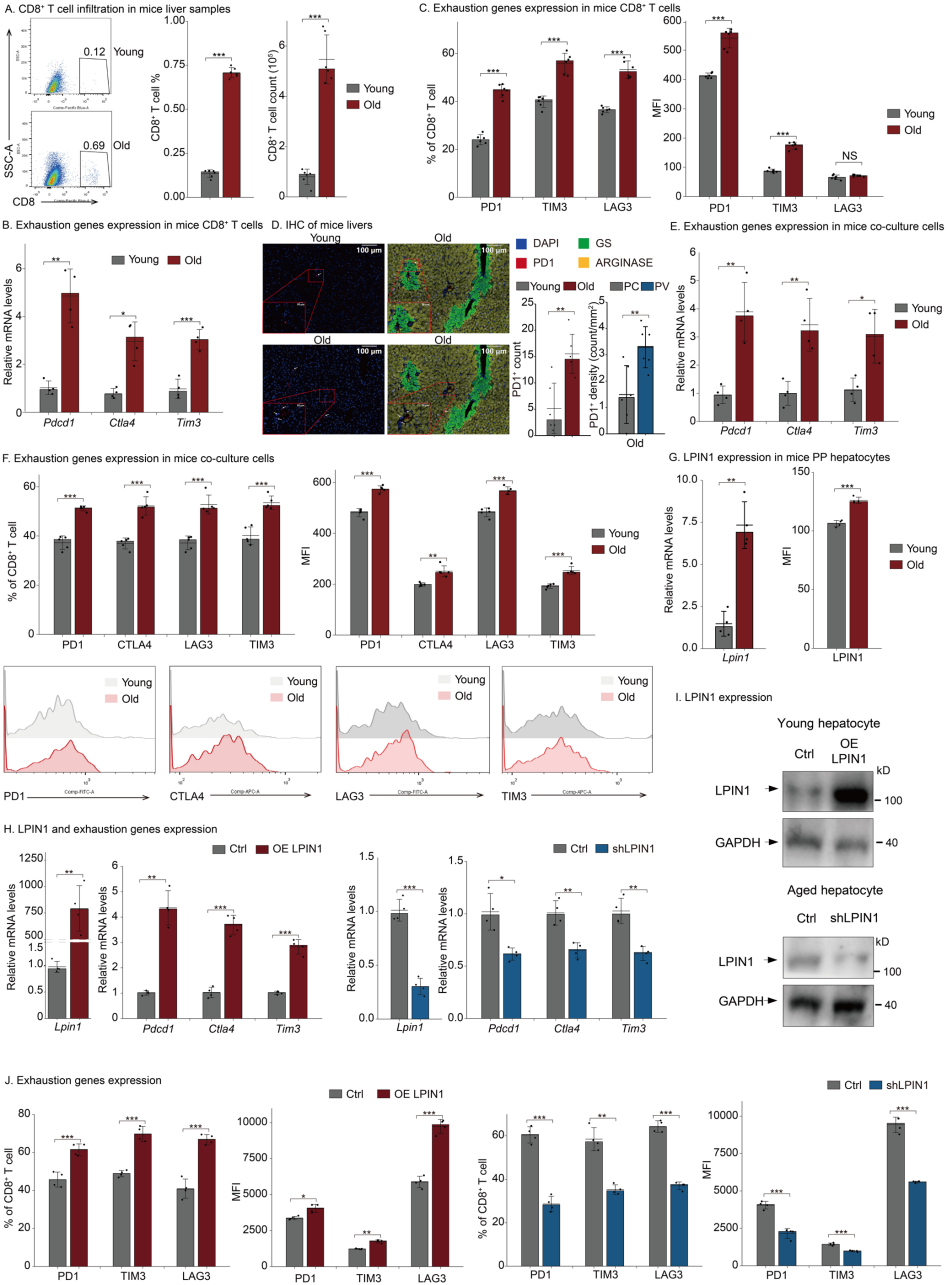
Old PP hepatocytes promote CD8^+^ T cell exhaustion via LPIN1 in mouse liver. (A–C) Livers in young (2 months) or aged (18 months) C57BL/6 mice were collected and liver‐infiltrated CD8^+^ T cells (*n* = 6 for each group) (A), mRNA of exhaustion genes (*n* = 4 for each group) (B), percentage and MFI of exhaustion proteins (*n* = 6 for each group) (C) were measured by FACS analysis or qRT‐PCR. (D) Young and old mice liver samples were subjected to multiplex immunohistochemistry for DAPI, PD1, GS (CV region marker), and ARGINASE (PV region marker). Each dot represents a section in a slide (*n* = 6 for each group). Bar, 100 μm (10×) and 20 μm (40X). (E, F) PP hepatocytes were sorted from livers of young or aged mice. Mouse naïve CD8^+^ T cells were enriched from C57BL/6 mice and stimulated with anti‐mouse CD3/CD28 antibodies two times every 2 days. Then hepatocytes were co‐cultured with CD8^+^ T cells and stimulated with anti‐mouse CD3/CD28 antibodies two times. mRNA of exhaustion genes (*n* = 4 for each group) (E), percentage and MFI of exhaustion proteins (*n* = 5 for each group) (F) were measured by qRT‐PCR or FACS analysis. (G) PP hepatocytes from young and old mice liver were sorted and mRNA and MFI of LPIN1 were measured by qRT‐PCR or FACS analysis (*n* = 4 for each group). (H–J) Hepatocytes from young mice were transfected with control or LPIN1 overexpression lentiviruses. Hepatocytes from aged mice were transfected with control or shLPIN1 lentiviruses. Then cells were co‐cultured with mouse CD8^+^ T cells for 4 days. *n* = 4 for each group. mRNA of exhaustion genes (H), percentage and MFI of exhaustion proteins (J) in CD8^+^ T cells were measured. mRNA (H) and protein (I) levels of LPIN1 in hepatocytes were measured by qRT‐PCR or western blot. (A–J) Statistical significance is tested using the two‐tailed unpaired *t*‐test with heteroscedasticity. NS *p* ≥ 0.05, *p < 0.05, **p < 0.01, ***p < 0.001.

To investigate whether aged livers contain increased numbers of CD8^+^ Tex, we examined the abundance and phenotype of CD8^+^ T cells in young and aged mouse livers. Flow cytometric and qRT‐PCR analysis revealed increased CD8^+^ T cell infiltration and a more pronounced exhausted phenotype in the aged group (Figure 6A–C). To further determine the spatial localization of CD8^+^ Tex cells, we performed immunohistochemistry (IHC) and observed a greater number of PD1‐positive cells in aged livers, with a marked enrichment in the PV region compared to the CV region (Figure 6D). To test whether PV‐zone PP hepatocytes in aged liver contribute to CD8^+^ T cell exhaustion, we conducted co‐culture experiments. CD8^+^ T cells co‐cultured with aged PP hepatocytes exhibited higher exhaustion levels compared to those cultured with young PP hepatocytes (Figure 6E,F). Our earlier transcriptomic analysis identified an upregulation of the triglyceride synthesis pathway in PP hepatocytes that positively correlated with T cell exhaustion; we therefore focused on LPIN1, the most significantly upregulated gene in this pathway (Table S2), for further validation. LPIN1 expression was confirmed to be elevated in aged PP hepatocytes (Figure 6G). Functionally, overexpression of LPIN1 in young hepatocytes promoted CD8^+^ T cell exhaustion, whereas LPIN1 knockdown in aged hepatocytes attenuated this effect (Figure 6H–J).

**FIGURE 7:**
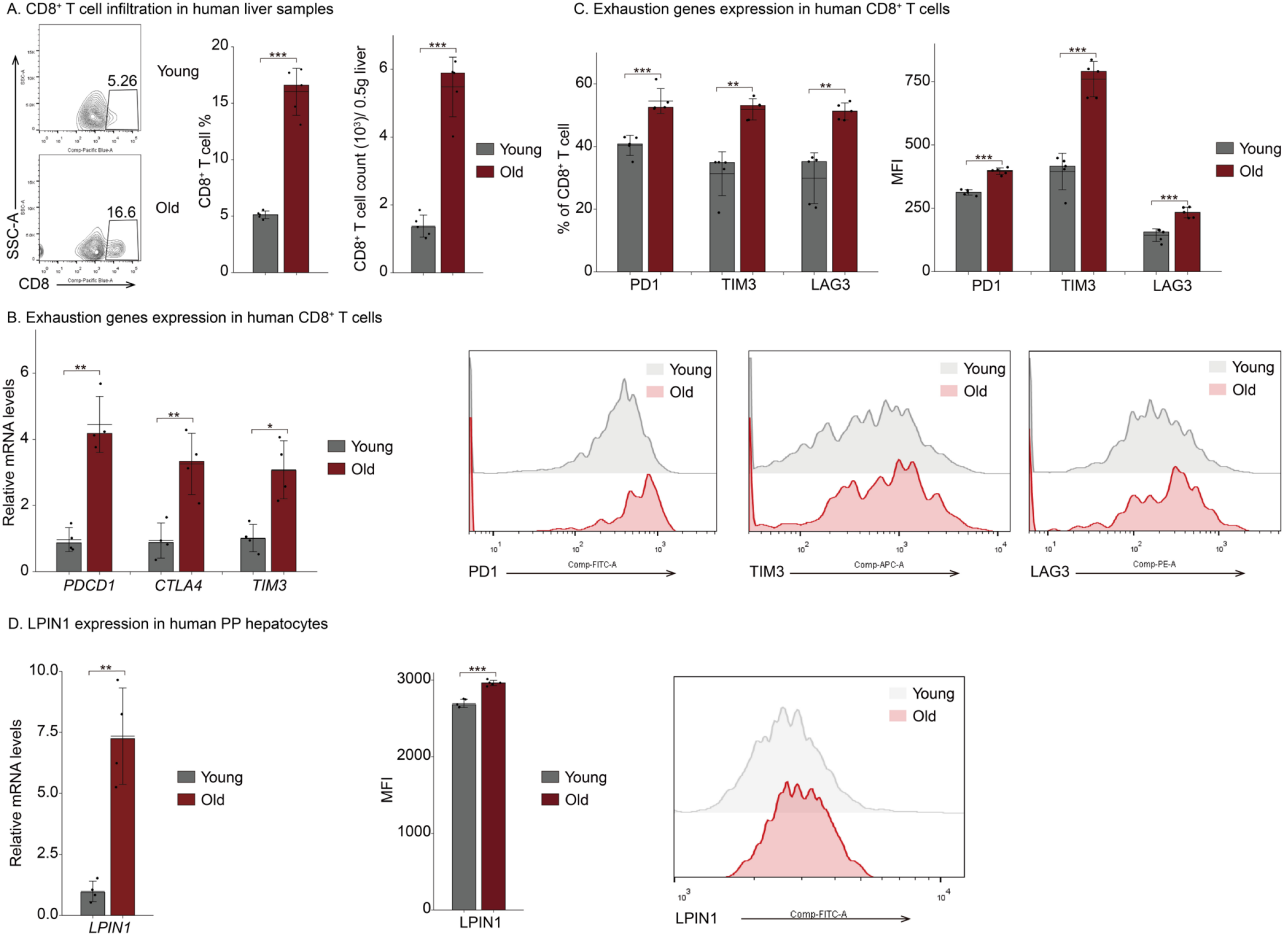
Detecting infiltration and exhaustion status of CD8^+^ T cells in young and old human livers. (A–C) Livers in young (< 65 years old) or aged (> 65 years old) humans were collected and liver‐infiltrated CD8^+^ T cells (*n* = 5 for each group) (A), mRNA of exhaustion genes (*n* = 4 for each group) (B), percentage and MFI of exhaustion proteins (*n* = 5 for each group) (C) were measured. (D) PP hepatocytes were sorted from livers of young or aged humans. mRNA (*n* = 4 for each group) and MFI (*n* = 5 for each group) of LPIN1 were measured. (A–D) Statistical significance is tested using the two‐tailed unpaired *t*‐test with heteroscedasticity. *p < 0.05, **p < 0.01, ***p < 0.001.

To further enhance the generalizability and clinical relevance of these findings, we performed validation studies using healthy human samples. We examined the abundance and phenotype of CD8^+^ T cells in liver tissue from young and aged individuals. Consistent with our mouse data, aged human livers exhibited increased CD8^+^ T cell infiltration and a more pronounced exhausted phenotype (Figure 7A–C). Moreover, LPIN1 expression was confirmed to be upregulated in aged PP hepatocytes (Figure 7D). Collectively, these results suggest that aged PP hepatocytes may promote CD8^+^ T cell exhaustion through upregulation of LPIN1 expression.

## 3 Discussion

Immune cells in the liver play a critical role in maintaining hepatic inflammatory homeostasis and clearing foreign pathogens and damaged cells. However, the dynamic changes and functional alterations of these immune cells during aging remain poorly understood. Our study delineated the aging landscape of hepatic immune cells using single‐cell/single‐nuclei transcriptomic and spatial transcriptomic profiling. Notably, we identify the most pronounced age‐related changes in T cells, characterized by the accumulation of exhausted T cells. Combining spatial and single cell analysis, we systematically investigated both T cell intrinsic and environmental mechanisms underlying T cell exhaustion. From an intrinsic perspective, aging drives a unique exhaustion program marked by upregulated expression of exhaustion‐associated genes (e.g., *Tox*, *Ptpn22*, *Lax1*) and downregulated memory/stemness‐related genes (e.g., *Il7r*, *Lef1*). From a microenvironmental perspective, exhausted T cells are specifically enriched in the PV zone, where PP hepatocytes may promote T cell exhaustion through LPIN1 up‐regulation.

T cells exhibit the most pronounced alterations among hepatic immune cell populations during liver aging, manifested by the accumulation of exhausted T cells. As critical effectors responsible for eliminating damaged cells, virus‐infected cells, and neoplastic cells in the liver, T cell exhaustion may elevate the risk of hepatic pathologies. Our multi‐model investigation across murine and human disease cohorts reveals a positive correlation between T cell exhaustion and the progression of liver diseases, suggesting that targeting exhausted T cells could represent a novel therapeutic strategy for hepatic disorders. However, a recent study in NASH murine models demonstrates that PD‐1 blockade antibodies—designed to reverse T cell exhaustion—paradoxically compromised anti‐tumor immune surveillance, resulting in elevated hepatocarcinogenesis rates (Pfister et al. 2021). These findings underscore the need for further mechanistic studies to clarify the causal relationship between T cell exhaustion and liver disease pathogenesis, particularly in the context of balancing therapeutic efficacy against potential oncogenic risks.

Our mechanistic investigation of T cell exhaustion in aged livers reveals that beyond intrinsic T cell transcriptional changes, metabolic alterations in hepatocytes critically contribute to the exhaustion process. We demonstrated through gain‐ and loss‐of‐function experiments that upregulation of LPIN1, a key regulator of triglyceride synthesis in PP hepatocytes, promotes T cell exhaustion. However, a limitation of our study is that only male mice were used and the precise mechanism by which LPIN1 exerts this pro‐exhaustion effect, and whether targeting LPIN1 could mitigate the impact of T cell exhaustion on the aged liver, remain to be elucidated and warrant further investigation.

## 4 Method

### 4.1 Ethics Statement

Young and aged mice of the C57BL/6J strain were housed in the Department of Experimental Animal Science, Peking University Health Science Center. All mice were housed in standard specific pathogen‐free conditions. All animal procedures were approved by the Institutional Animal Care and Use Committee (IACUC) of Peking University. The acquisition and use of clinical specimens received explicit authorization from the Ethics Committee at Peking University Shougang Hospital (Approval No. SGYYZ202103), with written consent documentation secured from all donors prior to participation.

### 4.2 Animals

C57BL/6 mice were purchased from Vital River Laboratory Animal Technology. Young (2 months) or aged mice (18 months) C57BL/6 mice were defined by age. All of the mice were maintained in pathogen‐free facilities and used strictly in accordance with the protocols approved by the IACUC of Tsinghua University. The study is compliant with all of the relevant ethical regulations regarding animal research. Sex was not considered in the study design and analysis since this study was not designed to detect sex differences. Only male mice were used for this study and all mouse data were collected from male mice.

### 4.3 Primary Hepatocyte Isolation and Culture

Young or aged mice were anesthetized according to previous methods (Bai et al. 2021). To obtain primary hepatocytes, liver tissues were harvested from male mice. A perfusion procedure was carried out via the portal vein using 50 mL of pre‐warmed HBSS (37°C, Corning) containing 1 mM EGTA and 5.5 mM glucose, delivered at a flow rate of 11 mL/min. This was followed by enzymatic digestion with 40 mL of HBSS‐based collagenase IV solution (Sigma, 0.25 mg/mL) supplemented with 5 mM CaCl_2_ and 5.5 mM glucose. Following dispersion, the hepatocyte suspension was centrifuged at 500 rpm for 2–5 min to sediment the cells. After two washing steps with M199 medium (MacGene, Beijing), the cell pellet was resuspended in attachment medium consisting of M199 supplemented with 0.2% BSA, 2% FBS, and 1% penicillin–streptomycin. The cells were then plated onto culture dishes pre‐coated with 0.1% gelatin and maintained at 37°C in a 5% CO_2_ incubator. After 4 h of incubation to allow attachment, the medium was replaced with fresh culture medium, and the cells were kept in culture for subsequent experimental analyses.

### 4.4 Cell Lines

The 293T cells were purchased from the American Type Culture Collection and cultured in DMEM medium (macgene, CM10013). 293T cells have been authenticated and were tested for mycoplasma contamination. Furthermore, we have authenticated the cell line by using a short tandem repeat profiling method according to the reported protocol.

### 4.5 DNA Constructs and Lentivirus Production

Fragments for mouse Lpin1 overexpression were constructed into pCHN1 vector. shRNA fragments were cloned into pLKO.1‐TRC. shRNA sequences were designed (F:CCGGGCCGTGTCATATCAGCAATTTCTCGAGAAATTGCTGATATGACACGGCTTTTTG, R:AATTCAAAAAGCCGTGTCATATCAGCAATTTCTCGAGAAATTGCTGATATGACACGGC) and cloned downstream of the U6 promoter within the lentiviral vector pLKO‐GFP, using a non‐targeting shRNA sequence as a control. Lentivirus was produced in HEK293T cells by co‐transfecting the above plasmids together with pMD2.G and psPAX2. Culture supernatants were collected and concentrated by ultracentrifugation at 20000 rpm for 2.5 h.

### 4.6 Isolation and Stimulation of Primary CD8 ^+^ T Cells

Mouse CD8^+^ T cells were isolated from mouse spleens using a biotin selection kit. CD8^+^ T cells and mouse P14 CD8^+^ T cells were stimulated with 3.5 μgmL^−1^ anti‐CD3 and 1 μgmL^−1^ anti‐CD28 antibodies. CD8^+^ T cells were cultured in RPMI‐1640 medium (macgene, CM10041) with 100 U/mL IL‐2. For in vitro exhaustion, CD8^+^ T cells were stimulated four times with 2 days' interval.

### 4.7 Flow Cytometry

For surface staining, cells were harvested and stained with antibodies at the recommended dilution prior to Flow Cytometry analysis. For intracellular protein LPIN1, cells were harvested and washed, fixed with eBioscience IC Fixation Buffer (00‐8222‐49), permeabilized with eBioscience Permeabilization Buffer (00‐8333‐56), and stained according to the manufacturer protocol. Stained cells were washed prior to FACS analysis. BD FACSuite Flow Cytometry Software was used for FACS signal collection. Positive events were determined by isotype control gating for each antibody. Cells were first gated using FSC/SSC characteristics to exclude debris, followed by gating FSC‐A and FSC‐H, then SSC‐A and SSC‐H to eliminate nonsinglets. Then target cells were gated to the population of interest by specific stain. Data analysis was carried out using FlowJo.

### 4.8 Multiplex Immunohistochemistry

A multiplex immunofluorescence staining approach was employed to sequentially detect three protein markers: PD1 (CST84651), Glutamine Synthetase (ab176562), and Arginase‐1 (16001‐1‐AP). The primary antibodies were applied in successive rounds: PD1 at a 1:100 dilution with overnight incubation at 4°C, followed by Glutamine Synthetase (1:1000, 30 min at room temperature) and Arginase‐1 (1:1000, 30 min at room temperature). Each cycle included incubation with HRP‐conjugated secondary antibodies and tyramide signal amplification (TSA) for fluorescence visualization. To prevent cross‐reactivity between staining rounds, microwave‐assisted antigen retrieval was performed after each TSA step using EDTA for PD1 and sodium citrate buffer for both Glutamine Synthetase and Arginase‐1. After completing the labeling of all target proteins, cell nuclei were counterstained with DAPI (Sigma‐Aldrich).

### 4.9 scRNA‐Seq Library Preparation and Sequencing

scRNA‐seq libraries were prepared using the 10× Genomics Chromium platform with the Single Cell 3′ Library & Gel Bead Kit v3.1 (1000121) and Single Cell G Chip Kit (1000120). Cell suspensions containing 300–600 viable cells/μL (quantified by Count Star viability analysis) were processed using the Chromium Controller to create gel bead‐in‐emulsion (GEM) partitions following the recommended workflow. Briefly, single cells were resuspended in phosphate‐buffered saline (PBS) supplemented with 0.04% bovine serum albumin (BSA), with approximately 12,000 cells loaded per microfluidic channel, yielding an anticipated recovery rate of ~10,000 cells per sample. Cellular lysis and subsequent RNA barcoding were achieved through GEM‐based reverse transcription. cDNA synthesis was conducted in a S1000TM Touch Thermal Cycler (Bio‐Rad) using the following thermal profile: 45 min at 53°C, 5 min at 85°C, followed by 4°C hold. Amplified cDNA products underwent quality control verification using the Agilent 4200 (service provided by CapitalBio Technology, Beijing). The constructed libraries were subsequently sequenced on an Illumina NovaSeq 6000 platform, employing a paired‐end 150 bp (PE150) strategy, with a sequencing depth of at least 100,000 reads per cell. This sequencing process was carried out by CapitalBio Technology in Beijing.

### 4.10 snRNA‐Seq Library Preparation and Sequencing

The mouse liver was placed in 1 mL lysis buffer (LB) and homogenized using a tissue homogenizer. The homogenate was then lysed on ice for 1–10 min. A 40 μm cell strainer was used to filter out debris, and the filtrate was centrifuged at 4°C and 500 *g* for 5 min. After removing the supernatant, 300 μL of LB solution was added to the sediment, and the nucleus was resuspended by pipetting. The suspension was transferred to a new centrifuge tube, and 300 μL of resuspension buffer (RB) was added, followed by thorough mixing. Next, 600 μL of PB1 solution was slowly added to the bottom of the tube to form a layer. Similarly, 600 μL of PB2 solution was slowly added to the bottom of the tube to form another layer. The mixture was then centrifuged at 4°C and 4000 *g* for 20 min, with the nucleus located at the interface between PB1 and PB2. A volume of 150–300 μL of the nucleus layer was transferred to 1 mL of RB solution, mixed thoroughly, and filtered through a 40 μm cell strainer. The filtrate was centrifuged at 4°C and 500 *g* for 5 min. After removing the supernatant, the nucleus sediment was resuspended in 100 μL of EB solution. Subsequent steps were carried out following the standard scRNA‐seq workflow. Library preparation and sequencing of the snRNA‐seq were performed by CapitalBio Technology in Beijing. The young and old groups were processed in separate batches, making it impossible to separate batch effects from differences caused by aging between the two groups.

### 4.11 Stereo‐Seq Library Preparation and Sequencing

Fresh mouse liver tissue specimens were adhered to BGI‐manufactured Stereo‐seq spatial transcriptomics chips through brief thermal conditioning at 37°C for 3 min. Subsequent fixation involved immersion in chilled absolute methanol (−20°C) for 40 min prior to initiating library construction workflows. For morphological validation, select sections underwent nuclear counterstaining with nucleic acid dye (Thermo Fisher Scientific, Q10212), with fluorescent micrographs captured using a Motic Custom PA53 FS6 microscope system. These quality‐controlled specimens were subsequently processed through FITC‐channel spatial barcoding workflows for transcriptome capture.

The tissue sections were initially treated with a buffer solution containing 0.1× SSC (Thermo, AM9770) and 0.05 U/mL RNase inhibitor (NEB, M0314L) to prepare them for subsequent steps. Permeabilization was achieved by immersing the sections in a solution containing 0.1% pepsin (Sigma, P7000) in 0.01 M HCl, followed by incubation at 37°C for 5 min. After this step, the sections were again washed with the same buffer containing RNase inhibitor. The RNA released from the permeabilized tissue was captured by DNA nanoballs (DNBs) and subjected to reverse transcription. This process utilized SuperScript II Reverse Transcriptase (Invitrogen, 18064‐014) in a reaction mixture containing 10 U/mL reverse transcriptase, 1 mM dNTPs, 1 M betaine, 7.5 mM MgCl_2_, 5 mM DTT, 2 U/mL RNase inhibitor, 2.5 mM Stereo‐seq TSO, and 1× First‐Strand buffer. The reverse transcription was carried out overnight at 42°C. Following this, the tissue sections underwent two washes with 0.1× SSC buffer and were then treated with Tissue Removal buffer (comprising 10 mM Tris–HCl, 25 mM EDTA, 100 mM NaCl, and 0.5% SDS) at 55°C for 10 min to facilitate tissue digestion. The cDNA‐containing chips were subsequently treated with a cDNA Release Mix (comprising cDNA Release Enzyme and buffer) overnight at 55°C. Finally, the cDNA was purified using VAHTSTM DNA Clean Beads at a 0.8× ratio to ensure high‐quality cDNA recovery.

cDNA amplification was performed using KAPA HiFi HotStart DNA Polymerase Master Mix (Roche, KK2602) supplemented with 0.8 μM cDNA‐specific amplification primers. Thermal cycling parameters were configured with the following profile: initial denaturation at 95°C for 5 min; 15 amplification cycles consisting of denaturation at 98°C for 20 s, primer annealing at 58°C for 20 s, and strand extension at 72°C for 3 min per cycle; concluding with a terminal extension phase at 72°C for 5 min to ensure complete product elongation.

DNA quantification of PCR products was performed using the Qubit dsDNA Assay Kit (Thermo, Q32854). For library preparation, 20 ng of purified DNA underwent fragmentation via incubation with custom‐formulated Tn5 transposase at 55°C for 10 min, with enzymatic activity terminated through the addition of 0.02% SDS solution followed by gentle mixing at 37°C for 5 min. Amplification reactions were conducted in 100 μL volumes containing 25 μL of fragmented DNA, 1× concentration of KAPA HiFi HotStart Ready Mix, 0.3 μM Stereo‐seq‐Library‐F primer, 0.3 μM Stereo‐seq‐Library‐R primer, and nuclease‐free water to reach the final volume. Thermal cycling conditions comprised an initial denaturation at 95°C for 5 min, followed by 13 cycles of 98°C for 20 s, 58°C for 20 s and 72°C for 30 s, concluding with a final extension at 72°C for 5 min. Amplified libraries underwent sequential purification using AMPure XP magnetic beads with size selection ratios of 0.63 and 0.153, followed by DNB generation through standard protocols. Final sequencing was executed on the MGI DNBSEQ‐Tx platform utilizing a paired‐end 150 bp configuration.

### 4.12 scRNA‐Seq and snRNA‐Seq Raw Data Processing

The original FASTQ files were aligned to the mm10 reference genome using the cellranger count function in cellranger 7.0.1 (10× Genomics Cell Ranger v7.0.1). For samples O2 and Y2, the parameter ‐force‐cells was explicitly set to 11,600 and 26,200, respectively, while default values were applied for other samples. The three output files generated by this pipeline were subsequently imported into Seurat 5.2.1 (Hao et al. 2024) for downstream analysis.

### 4.13 Stereo‐Seq Raw Data Processing

Sequencing data acquisition was performed on the MGI DNBSEQ‐Tx platform, generating paired‐end reads with embedded spatial identifiers. Read 1 contained dual oligonucleotide barcodes (CID: positions 1–25; MID: positions 26–35), while Read 2 carried cDNA sequences. Primary processing involved cross‐referencing CID sequences against predefined spatial coordinates derived from initial sequencing rounds, permitting single nucleotide mismatches to compensate for technical artifacts. Quality filtration excluded reads containing ambiguous bases (N) in MID regions or those exceeding two positions with Phred scores below 10. Validated CID‐MID combinations were integrated into FASTQ headers as metadata tags. Sequence alignment was executed through the STAR (Dobin et al. 2013) aligner against the reference genome mm10, retaining only uniquely mapped reads with mapping quality scores (MAPQ) exceeding 10 for gene annotation. Molecular deduplication consolidated UMIs sharing identical CID‐spatial coordinates and gene loci, incorporating single‐nucleotide tolerance to mitigate amplification biases. Final output generated a spatially resolved expression matrix encoding CID‐anchored transcriptional profiles. Bin200 was chosen as a spot.

### 4.14 Quality Control of scRNA‐Seq, snRNA‐Seq and Stereo‐Seq

#### 4.14.1 scRNA‐Seq Data QC

For each sample, two metrics were calculated to filter low‐quality cells and doublets: (1) mitochondrial gene UMI percentage and (2) total number of detected genes. Cells with mitochondrial UMI > 10% or < 500 total detected genes were classified as low‐quality, while those with > 6000 detected genes were flagged as doublets and excluded. Samples Y2 and O2, which showed significantly lower gene counts compared to other group members, were also excluded to minimize intra‐group heterogeneity affecting inter‐group comparisons.

#### 4.14.2 snRNA‐Seq Data QC

A similar workflow was applied, but with adjusted thresholds: cells with mitochondrial UMI > 5%, < 500 total genes, or > 5000 genes (doublets) were excluded. Sample Yn1 was removed due to its abnormally high proportion of low‐quality cells compared to other samples in its group.

#### 4.14.3 Stereo‐Seq Data QC (bin200 Spot)

Spots were assessed using mitochondrial UMI percentage (> 5%) and total detected genes (< 200) to identify low‐quality spots. No upper limit was imposed on gene counts, as each spot (bin200 resolution) may encompass multiple cells. All low‐quality spots were excluded from downstream analyses.

### 4.15 scRNA‐Seq and snRNA‐Seq Clustering and Annotation

The expression matrix was processed through the standard workflow in Seurat v5.2.1 (Hao et al. 2024). Initial normalization using the NormalizeData function eliminated technical variations in total UMI counts per cell. Two thousand highly variable genes were identified through the FindVariableFeatures function, followed by feature scaling via ScaleData to mitigate dominance effects from highly expressed genes. Dimensionality reduction was performed through principal component analysis (PCA) to capture major biological variations. For scRNA‐seq datasets, raw counts were directly merged using the merge function, with downstream analyses conducted in PCA‐reduced space. snRNA‐seq datasets required integration through canonical correlation analysis (CCA), with subsequent analyses performed in CCA‐aligned dimensional space. Sub‐clustering of scRNA‐seq data employed Harmony integration, with downstream analyses conducted in Harmony‐reduced space. Cell clustering utilized the first 50 principal components through shared nearest neighbor (SNN) graph‐based partitioning. Two‐dimensional visualization was achieved via RunUMAP for cluster representation. Final cell type annotation leveraged either literature‐reported marker genes or DEGs identified through model‐based analysis of single‐cell transcriptomics (MAST).

### 4.16 Marker Genes Used in Annotation

#### 4.16.1 Major Cell Type Annotation of scRNA‐Seq Data

T cell (*Cd3e* ^+^, *Cd3d* ^+^, *Cd3g* ^+^) (Liu et al. 2024; Su, Kim, et al. 2021); NK (*Klrb1c* ^+^) (Liu et al. 2024); NKT (*Cd3e* ^+^, *Cd3d* ^+^, *Cd3g* ^+^, *Klrb1c* ^+^) (Shen et al. 2020); B cell (*Cd19* ^+^, *Cd79a* ^+^, *Ms4a1* ^+^) (Liu et al. 2024; Su, Kim, et al. 2021); neutrophil (*Retnlg* ^+^) (Su, Kim, et al. 2021); DC (*Flt3* ^+^) (Su, Kim, et al. 2021); macrophage (*Clec4f* ^+^, *Adgre1* ^+^) (Wen et al. 2021; Krenkel and Tacke 2017; Liang et al. 2022); endothelial cell (*Clec4g* ^+^, *Ptprb* ^+^) (Su, Kim, et al. 2021; Drexler et al. 2019).

#### 4.16.2 Major Cell Type Annotation of snRNA‐Seq Data

PP hepatocyte (*Sds* ^+^, *Cyp2f2* ^+^) (Nikopoulou et al. 2023; Bravo González‐Blas et al. 2024); PC hepatocyte (*Glul* ^+^, *Cyp2e1* ^+^) (Bravo González‐Blas et al. 2024); endothelial cell (*Dpp4* ^+^) (Nikopoulou et al. 2023); hepatic stellate cell (*Reln* ^+^) (Su, Kim, et al. 2021); immune cell (*Ptprc* ^+^) (Liu et al. 2024).

#### 4.16.3 Subcluster Annotation of T Cell

CD8^+^ T cells: naïve state (CD8^+^ Tn, *Lef1* ^+^, *Sell* ^+^, *Il7r* ^+^) (Liu et al. 2024; Technology, C. S, n.d.); central memory state (CD8^+^ Tcm, *Sell* ^+^, *CD44* ^+^, *Il7r* ^+^) (Technology, C. S, n.d.); effector memory state (CD8^+^ Tem, *CD44* ^+^, *Il7r* ^+^) (Technology, C. S, n.d.); exhausted state (CD8^+^ Tex, *Pdcd1* ^+^, *Tigit* ^+^, *Lag3* ^+^, *Tox* ^+^) (Mogilenko et al. 2021).

CD4^+^ T cells: naïve state (CD4^+^ Tn, *Lef1* ^+^, *Sell* ^+^, *Il7r* ^+^) (Liu et al. 2024; Technology, C. S, n.d.); effector memory state (CD4^+^ *Tem*, *CD44* ^+^, *Il7r* ^+^) (Technology, C. S, n.d.); regulator state (CD4^+^ Treg, *Foxp3* ^+^, *Il2ra* ^+^, *Ctla4* ^+^) (Ohkura and Sakaguchi 2020); exhausted state (CD4^+^ *Pdcd1* ^+^ T, *Pdcd1* ^+^, *Tigit* ^+^, *Lag3* ^+^, *Tox* ^+^) (Mogilenko et al. 2021).

Other T cells: CD4/CD8 negative Treg‐like T cell (DN Treg like, *Il2ra* ^+^).

#### 4.16.4 Subcluster Annotation of NK

Stress NK (*Hspa1a* ^+^, *Hspa1b* ^+^, *Hsph1* ^+^).

#### 4.16.5 Subcluster Annotation of NKT

Trafficking NKT (*Klf2* ^+^, *S1pr1* ^+^, *Sell* ^+^) (Shen et al. 2020); Active NKT (*Icos* ^+^) (Shen et al. 2020); *Cd200r1* ^+^ NKT (*Cd200r1* ^+^).

#### 4.16.6 Subcluster Annotation of B Cell

Naïve B (*Ighd* ^+^) (Biocompare, n.d.); Stress B (*Hspa1a* ^+^,*Hspa1b* ^+^); age‐associated B cell like B cell type 1 (ABC like B1, *Zbtb32* ^+^, *Fcrl5* ^+^, *Tbx21* ^+^, *Cr2* ^−^, *Fcer2a* ^−^) (Mogilenko et al. 2021; Hao et al. 2011); age‐associated B cell like B cell type 2 (ABC like B2, *Fcrl5* ^+^, *Cr2* ^−^, *Fcer2a* ^−^, *Cd93* ^−^, *Fcer1g* ^−^, *Spn* ^−^) (Mogilenko et al. 2021; Hao et al. 2011).

#### 4.16.7 Subcluster Annotation of Neutrophil

*Fnip2* ^+^ Neu (*Fnip2* ^+^, *Ccrl* ^+^); *Cxcl3* ^+^ Neu (*Cxcl3* ^+^); *Ngp* ^+^ Neu (*Fgd4* ^+^, *Ngp* ^+^, *Camp* ^+^).

#### 4.16.8 Subcluster Annotation of DC

*Grm8* ^+^ pDC (*Bst2* ^+^, *Siglech* ^+^, *Grm8* ^+^) (Segure 2016); *Apod* ^+^ pDC (*Bst2* ^+^, *Siglech* ^+^, *Apod* ^+^) (Segure 2016); *Batf3* ^+^ cDC1 (*Batf3* ^+^) (Segure 2016); *Xcr1* ^+^ cDC1 (*Batf3* ^+^, *Xcr1* ^+^) (Segure 2016); cDC2 (*Cd209a* ^+^) (Liu et al. 2024).

#### 4.16.9 Subcluster Annotation of Macrophage

Macrophages derived from monocytes (*Cx3cr1* ^+^) (Krenkel and Tacke 2017): *Vcan* ^+^ Mo‐Mac (*Vcan* ^+^); *Dusp16* ^+^ Mo‐Mac (*Dusp16* ^+^); *Stxbp6* ^+^ Mo‐Mac (*Stxbp6* ^+^); *Cxcl2* ^+^ Mo‐Mac (*Cxcl2* ^+^); *Cxcl9* ^+^ Mo‐Mac (*Cxcl9* ^+^).

Other macrophages: Kupffer cell (*Clec4f* ^+^, *Vsig4* ^+^) (Nikopoulou et al. 2023; Su, Kim, et al. 2021); *Cxcl9* ^+^ Mac (*Cxcl9* ^+^); *Spp1* ^+^ Mac (*Spp1* ^+^).

### 4.17 Cell Enrichment (Chu et al. 2024)

To determine whether specific cell types exhibit enrichment in the old group, cellular enrichment analysis was performed using the chi‐square test. For cell type X, all immune cells were categorized into four comparative groups: X cells in the old group, X cells in the young group, non‐X cells in the old group, and non‐X cells in the young group. Quantitative cell counts from these four categories were statistically analyzed through the chi‐square test. The ratio of observed to expected values (Ro/e) was calculated as the number of X cells observed in the old group divided by the theoretical number of X cells predicted for the old group under the null hypothesis, providing a metric to quantify enrichment level.

### 4.18 DEGs Analysis

Differential gene expression analysis was conducted using the FindMarkers function in Seurat v5.2.1 (Hao et al. 2024) with the MAST algorithm. For identifying senescence‐associated genes across major immune cell populations, comparative analyses were performed between old and young groups within each immune cell category, applying thresholds of |avg_log2FC| > 1 and *p*_val_adj < 0.05 to define significantly DEGs. In the analysis of PP hepatocytes in the old group, three distinct comparisons were established: old PP hepatocytes vs. old PC hepatocytes in snRNA‐seq data, PV zone spatial spots vs. CV zone spatial spots within the old group of Stereo‐seq data, and old PV zone spatial spots vs. young PV zone spatial spots in Stereo‐seq data. For these PP hepatocyte comparisons, DEG identification employed more lenient criteria of |avg_log2FC| > 0.25 with *p*_val_adj < 0.05.

### 4.19 Bhattacharyya Distance Computation

The Bhattacharyya distance, a measure of similarity between two distributions, is effective in quantifying differences between two clusters in high‐dimensional space. To compute this metric, the distdimscr software (version 0.0.0.9000), which is available at https://github.com/arc85/distdimscr was utilized, was used. To enhance computational efficiency, a random sampling approach was implemented: for each major immune cell population, 250 cells were randomly selected for analysis, and this sampling process was repeated 100 times.

### 4.20 Enrichment Analysis of DEGs

DEGs were categorized into upregulated and downregulated groups for separate enrichment analyses, which were performed using Metascape (Zhou et al. 2019) (https://metascape.org/gp/). The pathways subjected to enrichment analysis were sourced from five databases: GO Molecular Functions, GO Biological Processes, GO Cellular Components, Reactome Gene Sets, and KEGG Pathway.

### 4.21 Pathway and Signature Gene Scoring

Pathway scoring was performed using the AddModuleScore function in Seurat v5.2.1 (Hao et al. 2024). T cell state‐related pathways (quiescence, proliferation, cytotoxicity, progenitor exhaustion, terminal exhaustion, senescence) were sourced from TCellSI v0.1.0 (Yang et al. 2024). The apoptosis activation pathway was derived from the literature (Jourdan et al. 2009), while the TGFβ response pathway (HALLMARK_TGF_BETA_SIGNALING) was obtained from the MSigDB database. Marker genes for cell types were identified through lineage‐internal differential expression analysis: *Ngp* ^+^ Neu vs. remaining neutrophil subtypes, *Spp1* ^+^ Mac vs. other macrophage subtypes, *Apod* ^+^ pDC vs. other DC subtypes, naïve B cells versus other B cell subtypes, and T/NK/NKT cell subsets vs. their respective remaining populations. Marker genes comprised the top 30 highly expressed genes per cell type and manually curated genes from literatures (Bravo González‐Blas et al. 2024; Su, Yang, et al. 2021) for endothelial cells and hepatocytes. All marker gene sets exhibited no overlap between cell classes or layer scoring gene sets. Replicative senescence (GO:0090399), cellular senescence (GO:009038), and SASP pathways were curated from MSigDB and the literature (Suryadevara et al. 2024). Metabolic pathways—amino acid synthesis (mmu00290, mmu00220, mmu00400), insulin signaling (mmu04910), steroid hormone synthesis (mmu00140), tyrosine metabolism (mmu00350), gluconeogenesis (R‐MMU‐70263), oxaloacetate metabolism (GO:0006107), triglyceride synthesis (GO:0019432), glucagon response (GO:0033762), alcohol response (GO:0097305), fatty acid synthesis (GO:0006633), and fatty acid degradation (GO:0009062)—were retrieved from KEGG and MSigDB.

### 4.22 Collection and Analysis of Public scRNA‐Seq Datasets

Liver single‐cell datasets from healthy young and old mice were selected from studies (Mogilenko et al. 2021; Almanzar 2020; Cai et al. 2023; Nikopoulou et al. 2023) for analysis. Quality control procedures followed the original study protocols with additional adjustments: The study (Cai et al. 2023) implemented a count threshold of < 10,000; The study (Mogilenko et al. 2021) applied a maximum count limit of 20,000; The study (Nikopoulou et al. 2023) excluded cells exceeding 20% mitochondrial gene content; The study (Almanzar 2020) supplemented droplet‐based data with a 50,000‐count ceiling and FACS‐sorted data with a 1 × 10^7^ count ceiling. Disease‐associated liver scRNA‐seq datasets from the studies (Xiong et al. 2019; Su, Kim, et al. 2021; Ramachandran et al. 2019; Li et al. 2024) underwent quality control according to their respective original publications.

### 4.23 Cell Differentiation Trajectory

Differentiation trajectory analysis of CD8^+^ T cells was performed using Monocle v2.26.0 (Qiu et al. 2017). Raw count matrices were converted into CellDataSet object, followed by library size normalization and dispersion estimation via the estimateSizeFactors function. DEGs across CD8^+^ T cell subsets were identified using the differentialGeneTest method, with the top 200 DEGs selected as input features. Trajectory reconstruction was executed through the DDRTree algorithm, establishing CD8^+^ Tn as the root state. Branch‐specific expression patterns in two exhaustion trajectories were analyzed using the BEAM function, with DEGs defined by a *q*‐value < 0.05 and detection in ≥ 5% of cells.

### 4.24 Stereo‐Seq Annotation

scRNA‐seq and snRNA‐seq data generated in this study were used as references after excluding stress B cells, DN Treg‐like cells, and stress NK populations. Spatial transcriptomic data from old samples were deconvolved using references from the old group, while young spatial data employed corresponding young‐group references. The first annotation approach utilized cell2location v0.1.3 (Kleshchevnikov et al. 2022) following its standard workflow. Mitochondrial genes were removed, followed by gene filtering with default parameters (cell_count_cutoff = 5, cell_percentage_cutoff2 = 0.03, nonz_mean_cutoff = 1.12). Reference signature matrices were constructed with sample‐level batch correction and trained for 1000 epochs. Spatial resolution modeling was performed using hyperparameters N_cells_per_location = 32 (determined based on murine hepatic nuclear density data from the literature (Hildebrandt et al. 2021)) and detection_alpha = 20, followed by 10,000‐epoch training. Cell abundance was quantified using the 5th percentile of posterior distributions as recommended. The second approach employed stereoscope v0.3.1 (Andersson et al. 2020), where input data were prepared via subsample‐data.py (max 500 cells per class) and deconvolution executed using stereoscope run with parameters ‐sce 50000, ‐n 5000, ‐ste 50,000, ‐stb 100, and ‐scb 100.

### 4.25 Layer Allocation

Spatial transcriptomics spots were scored for PV and CV zonation using previously reported marker genes (Xu et al. 2024), implemented via the AddModuleScore function in Seurat v5.2.1 (Hao et al. 2024). The final zonation score was calculated as CV signature score minus PV signature score. Within each sample, all spots were stratified into nine equal quantiles (layer1‐layer9) based on descending zonation scores, with CV regions defined as layers 1–3, intermediate zones as layers 4–6, and PV regions as layers 7–9.

## Author Contributions

Jie Cheng and Jiahua Lu conceived the experimental design. Zihao Zhao performed mice samples preparation for sequencing under the supervision of Jie Cheng. Jiahua Lu, and Wenxue Zhao performed bioinformatics analyses with the supervision of Jie Cheng and Cheng Li. Zhaoya Gao collected human samples. Yuqian Wang performed experiments. Jiahua Lu wrote the manuscript. Jie Cheng, Cheng Li, and Jin Gu supervised the research. All authors commented on the manuscript.

## Funding

This research was supported by National Science and Technology Major Project of the Ministry of Science and Technology of China (2025ZD0549900) to Jie Cheng. This work was supported by National Natural Science Foundation of China (32288102, 32025006) and National Key Research and Development Program of China (2021YFA1100300) to Cheng Li. This work was supported by Beijing Natural Science Foundation (7254382) and National Natural Science Foundation of China (32500758) to Yuqian Wang.

## Disclsoure

Lead Contact: For further information or to request access to materials, kindly reach out to the lead author, J.C., via email at jiecheng2024@hust.edu.cn.

## Conflicts of Interest

The authors declare no conflicts of interest.

## Supporting information


**Figure S1:** Quality control, clustering and annotation of scRNA‐seq and snRNA‐seq data. (A) Quality control of scRNA‐seq data. Red represents the old group, while gray denotes the young group. Cells were excluded if they met any of the following criteria: mitochondrial gene percentage > 10%, detected genes > 6000, or detected genes < 500. (B) Quality control of snRNA‐seq data. Red represents the old group, while gray denotes the young group. Cells were excluded if they met any of the following criteria: mitochondrial gene percentage > 5%, detected genes > 5000, or detected genes < 500. (C) UMAP plot of CD45^+^ sorted cells. Left, colors are assigned based on samples. Right, colors are assigned based by groups. (D) Marker genes of CD45^+^ sorted cells. Colors represent scaled average expression. Dot size represents the percentage of cells that express the gene. Marker genes of the same subcluster are highlighted with a box. (E) UMAP plot of unsorted cells before integration. Left, colors are assigned based on samples. Right, colors are assigned based by groups. (F) UMAP plot of unsorted cells after integration. Left, colors are assigned based on samples. Right, colors are assigned based by groups. (G) Marker genes of unsorted cells. Colors represent scaled average expression. Dot size represents the percentage of cells that express the gene. Marker genes of the same subcluster are highlighted with a box. (A–G) scRNA‐seq and snRNA‐seq data were generated in house.

**Figure S2:** Clustering and annotation of T cells, NK, NKT, and DC. (A) UMAP plot of T cells with cells colored by group. Upper, before integration. Bottom, after integration. (B) Marker genes of T cells. Colors represent scaled average expression. Dot size represents the percentage of cells that express the gene. Marker genes of the same subcluster are highlighted with a box. (C) UMAP plot of NK cells with cells colored by group. Upper, before integration. Bottom, after integration. (D) UMAP plot of NK cells with cells colored by cluster. (E) Marker genes of NK cells. Colors represent scaled average expression. Dot size represents the percentage of cells that express the gene. Marker genes of the same subcluster are highlighted with a box. (F) UMAP plot of NKT cells with cells colored by group. Left, before integration. Right, after integration. (G) UMAP plot of NKT cells with cells colored by cluster. (H) Marker genes of NKT cells. Colors represent scaled average expression. Dot size represents the percentage of cells that express the gene. Marker genes of the same subcluster are highlighted with a box. (I) UMAP plot of DCs with cells colored by group. Upper, before integration. Bottom, after integration. (J) UMAP plot of DCs with cells colored by cluster. (K) Marker genes of DCs. Colors represent scaled average expression. Dot size represents the percentage of cells that express the gene. Marker genes of the same subcluster are highlighted with a box. (A–K) scRNA‐seq data were generated in house.

**Figure S3:** Clustering and annotation of macrophages, B cells and neutrophils. (A) UMAP plot of macrophages with cells colored by group. Upper, before integration. Bottom, after integration. (B) UMAP plot of macrophages with cells colored by cluster.
(C) Marker genes of macrophages. Colors represent scaled average expression. Dot size represents the percentage of cells that express the gene. Marker genes of the same subcluster are highlighted with a box. (D) UMAP plot of B cells with cells colored by group. Upper, before integration. Bottom, after integration. (E) UMAP plot of B cells with cells colored by cluster. (F) Marker genes of B cells. Colors represent scaled average expression. Dot size represents the percentage of cells that express the gene. Marker genes of the same subcluster are highlighted with a box. (G) UMAP plot of neutrophils with cells colored by group. Upper, before integration. Bottom, after integration. (H) UMAP plot of neutrophils with cells colored by cluster. (I) Marker genes of neutrophils. Colors represent scaled average expression. Dot size represents the percentage of cells that express the gene. Marker genes of the same subcluster are highlighted with a box. (A–I) scRNA‐seq data were generated in house.

**Figure S4:** Difference of cell proportion between young and old groups and state analysis of T cells. (A) Proportion of the four most divergent subclusters. Ro/e, observed number in old group versus expected number in old group. Statistical significance is tested using the chi‐squared test. ***p < 0.001. (B–D) Split UMAP plot of the four most divergent subclusters. (A–D) scRNA‐seq data were generated in house. (E) Log_2_ fold change of cell proportion. Subclusters are sorted based on the log_2_ fold change of old group mean proportion versus young group mean proportion. Colors are assigned in order. (F) Subclusters proportion. Colors are consistent with those in the plot E. (G) Proportion of CD8^+^ Tex. Ro/e, observed number in old group versus expected number in old group. Statistical significance is tested using the chi‐square test. ***p < 0.001. (H) Split UMAP plot of CD8^+^ Tex. (E–H) scRNA‐seq data were collected from public databases (Mogilenko et al. 2021; Almanzar 2020; Cai et al. 2023; Nikopoulou et al. 2023). (I) Expression self‐attack genes (Dudek et al. 2021) in CD8^+^ T cells. Colors represent scaled average expression. Dot size represents the percentage of cells that express the gene. (J) *Icam1* (Dudek et al. 2021) expression in non‐immune cells. (K) TGFβ response and apoptosis activation scores in each state. Statistical significance is tested using the two‐tailed unpaired *t*‐test with heteroscedasticity. NS *p* ≥ 0.05, **p < 0.01. (L) UMAP plot of CD8^+^ Tex with cells colored by group. Left, before integration. Right, after integration. (I–L) scRNA‐seq data were generated in house.

**Figure S5:** Quality control of Stereo‐seq data and layer allocation. (A) Quality control of Stereo‐seq data. Red represents the old group, while gray denotes the young group. Spots were excluded if they met any of the following criteria: mitochondrial gene percentage > 5%, or detected genes < 200. (B) Layer allocation. The first three subplots represent the spatial distribution of region score, central vein (CV) zone marker gene *Cyp2e1*, and PV zone marker gene *Cyp2f2*, respectively. The color intensity indicates higher scores or expression levels in red and lower values in blue. The rightmost subplot shows the layer classification, with layers 1–9 representing the transition from CV zone to PV zone. (A, B) Stereo‐seq data were generated in house.

**Figure S6:** Density, proportion and signature scores distribution in layers. (A–C) The color gradient represents scaled density values, with red indicating higher values and blue indicating lower values. The numbers within each box show the raw (unscaled) density. Round to two decimal places only. A, cell density distribution of hepatocytes. B, cell proportion distribution of hepatocytes. C, cell density distribution of CD8^+^ T cells. (D) Signature scores of periportal (PP) and pericentral (PC) endothelial cells. Statistical significance is tested using the ANOVA. ***p < 0.001. (E) CD8^+^ Tex density and proportion in different layers. Statistical significance is tested using the two‐tailed unpaired t‐test with hetereoscedasticity. ***p < 0.001. (A–E) Stereo‐seq data were generated in house.

**Figure S7:** Signature scores distribution of CD8^+^ T cell subclusters in layers. (A) CD8^+^ Tex signature scores distribution in young group. Statistical significance is tested using the ANOVA. NS *p* ≥ 0.05. (B) Other CD8^+^ T cell subclusters signature scores distribution. Statistical significance is tested using the ANOVA. NS *p* ≥ 0.05, **p < 0.01, ***p < 0.001. (A, B) Stereo‐seq data were generated in house.

**Figure S8:** Specificity of signature score. (A) Violin plot of signature scores. scRNA‐seq data were generated in house. Corresponding subset is highlighted in red.

**Figure S9:** Spatial distribution of density. (A) Density distribution. Color represents density. In monochrome mode, brighter shades indicate higher density, while darker shades indicate lower density. “Merge” denotes the blending of colors corresponding to the densities of two subclusters. Stereo‐seq data were generated in house.
(B) Colocalization score of CD8^+^ Tex with hepatocytes. The blue histogram represents the distribution of permutation‐based colocalization scores (10,000 permutations), and the red dashed line indicates the mean observed colocalization score.

**Figure S10:** Scores of aging‐associated and metabolic pathways. (A) Aging associated pathway scores across zones. SASP, senescence‐associated secretory phenotype. Statistical significance is tested using the two‐tailed unpaired t‐test with hetereoscedasticity. NS *p* ≥ 0.05, **p < 0.01, ***p < 0.001. (B) Metabolic pathway scores across hepatocyte subclusters and zones. Statistical significance is tested using the two‐tailed unpaired *t*‐test with hetereoscedasticity. ***p < 0.001. (A, B) Stereo‐seq and snRNA‐seq data were generated in house.

**Figure S11:** Gating strategy used in FACS analysis for mouse cells. (A) Gating strategy to analyze mouse PP hepatocytes for LPIN1 expression. (B) Gating strategy to analyze mouse liver infiltrated CD8^+^ T cells for LAG3, TIM3, PD1, CTLA4 expression.

**Figure S12:** Gating strategy used in FACS analysis for human cells. (A) Gating strategy to analyze human PP hepatocytes for LPIN1 expression. (B) Gating strategy to analyze human liver infiltrated CD8^+^ T cells for LAG3, TIM3, PD1 expression.

**Table S1:** Pathway and signature genes.
**Table S2:** Upregulation of triglyceride anabolism‐related genes in old PP hepatocytes.

## Data Availability

Raw scRNA‐seq/snRNA‐seq data and processed data of this study can be downloaded at NCBI Gene Expression Omnibus (GEO) with accession number GSE327302 and GSE327303. Processed ST data of this study can be downloaded at Zenodo (https://zenodo.org/records/19524969). Public scRNA‐seq datasets were obtained from GEO under accession number GSE155006 (Mogilenko et al. 2021), GSE132042 (Almanzar 2020), GSE136103 (Ramachandran et al. 2019), GSE166504 (Su, Kim, et al. 2021), GSE129516 (Xiong et al. 2019), from figshare at https://doi.org/10.6084/m9.figshare.22332568 (Li et al. 2024), from EMBL‐EBI under accession number E‐MTAB‐12579 (Nikopoulou et al. 2023), and from GSA under accession number CRA010641 (Cai et al. 2023). The study used standard analysis methods and didn't develop new algorithms. The analysis code (R/Python) is available by contacting the lead researchers.
