# Supplementary figures and images for: Spatiotemporal Transcriptomics Characterizes Immune Microenvironment During Mouse Liver Aging
Source: Aging Cell. 2026 Apr 20;25(5):e70482. doi: 10.1111/acel.70482 (PMC13096584; doi:10.1111/acel.70482)

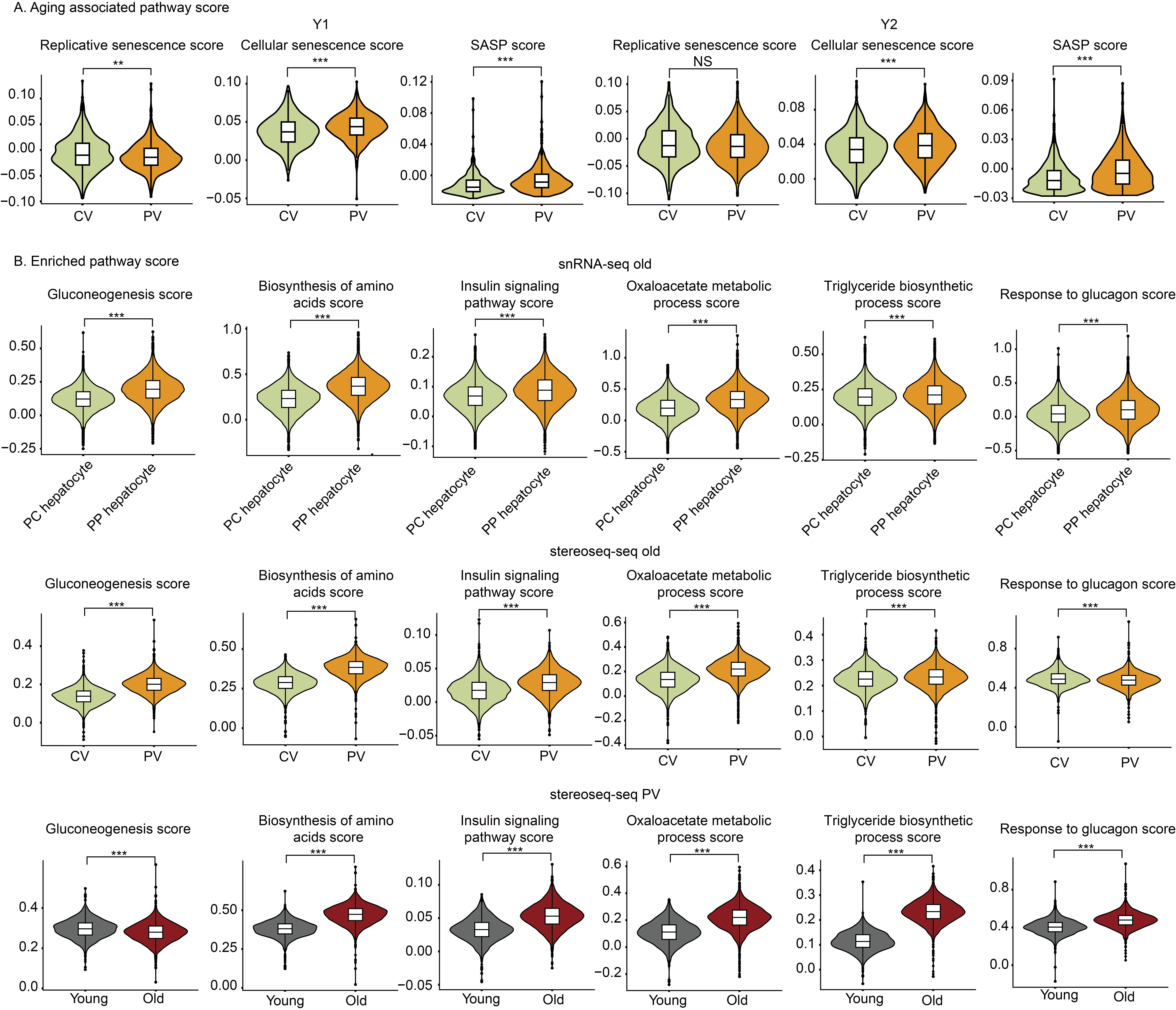

Supplement: Supplementary file 10 — Figure S10: Scores of aging‐associated and metabolic pathways. (A) Aging associated pathway scores across zones. SASP, senescence‐associated secretory phenotype. Statistical significance is tested using the two‐tailed unpaired t‐test with hetereoscedasticity. NS p ≥ 0.05, **p < 0.01, ***p < 0.001. (B) Metabolic pathway scores across hepatocyte subclusters and zones. Statistical significance is tested using the two‐tailed unpaired t‐test with hetereoscedasticity. ***p < 0.001. (A, B) Stereo‐seq and snRNA‐seq data were generated in house. [file ACEL-25-e70482-s002.jpg]
