# Supplementary material for: Spatiotemporal Transcriptomics Characterizes Immune Microenvironment During Mouse Liver Aging
Source: Aging Cell. 2026 Apr 20;25(5):e70482. doi: 10.1111/acel.70482 (PMC13096584; doi:10.1111/acel.70482)
Supplement: Supplementary file 13 — Table S1: Pathway and signature genes. Table S2: Upregulation of triglyceride anabolism‐related genes in old PP hepatocytes. [file ACEL-25-e70482-s003.pdf]

Supplemental Table 1. pathway and signature					
quiescence	*Btg2*	*Klf2*	*Foxp1*	*Runx1*	*Myc*
proliferation	*Mki67*	*Tyms*	*Ttk*	*Cks1b*	*Brip1*
	Gins2	*Cks2*	*Anln*	*Cd4*	*Cd8a*
	Cd8b	*Cd3g*	*Cd3d*	*Cd3e*	*Cd247*
	Cd8b1				
cytotoxicity	*Gzmb*	*Gzmh*	*Gzmk*	*Gzma*	*Tia1*
	Prf1	*Lamp1*	*Gnly*	*Faslg*	*Slamf7*
	Zap70	*Cd69*	*Tnf*	*Fasl*	
progenitor exhaustion	*Ctla4*	*Pdcd1*	*Hnf1a*	*Slamf6*	*Cd8a*
	Cd8b	*Tnf*	*Icos*	*Tnfsf14*	*Satb1*
	Eomes	*Id3*	*Tcf7*	*Cxcr3*	*Gzmk*
	Sell	*Il7r*	*Ccr7*	*Cxcl10*	*Cd44*
	Bcl6	*Cd27*	*Cd28*	*Cd38*	*Cd8b1*
terminal exhaustion	*Cxcl13*	*Entpd1*	*Prf1*	*Gzmh*	*Gzmb*
	Gzma	*Batf*	*Lyst*	*Batf*	*Tigit*
	Lag3	*Havcr2*	*Pdcd1*	*Prdm1*	*Rbpj*
	Slamf7	*Ccl4*	*Tnfsf10*	*Tbx21*	*Nfatc1*
	Hif1a	*Tnfrsf4*	*Cxcr6*	*Ptpn7*	*Ewsr1*
	Hla-Dpb1	*Hla-Dpa1*	*Ubxn1*	*Psmb9*	*Ly6e*
	Ccndbp1	*Irf9*	*Lcp2*		
senescence	*Cd3g*	*Cd3d*	*Cd3e*	*Cd247*	*Klrg1*
	B3gat1	*Tp53*	*Mapk14*	*Mapk8*	*Mapk1*
	Cdkn2a	*B3gat1*			
apoptosis activation	*Bax*	*Bak1*	*Bok*	*Bid*	*Bcl2l11*
	Bmf	*Bad*	*Bik*	*Hrk*	*Pmaip1*
	Bnip3	*Bnip3l*	*Bcl2l14*	*Bbc3*	*Bcl2l12*
	Bcl2l13	*Fas*	*Tnfrsf10b*	*Tnfrsf11b*	*Tnfrsf1a*
	Fadd	*Casp1*	*Casp2*	*Casp3*	*Casp4*
	Casp6	*Casp7*	*Casp8*	*Casp9*	*Casp14*
TGFβ response	*Ppp1ca*	*Nog*	*Cdkn1c*	*Fnta*	*Skil*
	Xiap	*Ifngr2*	*Hdac1*	*Slc20a1*	*Smad1*
	Bmpr2	*Rhoa*	*Smad7*	*Klf10*	*Tgif1*
	Smad3	*Hipk2*	*Cdk9*	*Smad6*	*Ncor2*
	Bmpr1a	*Map3k7*	*Bcar3*	*Ube2d3*	*Smurf2*
	Rab31	*Wwtr1*	*Smurf1*	*Ppp1r15a*	*Pmepa1*
	Trim33	*Arid4b*	*Acvr1*	*Apc*	*Bmp2*
	Ctnnb1	*Cdh1*	*Eng*	*Fkbp1a*	*Id1*
	Id2	*Id3*	*Junb*	*Furin*	*Serpine1*
	Ski	*Sptbn1*	*Tgfb1*	*Tgfbr1*	*Thbs1*

	Tjp1	*Ltbp2*	*Ppm1a*		
Apod⁺ pDC	*Apod*	*Hrh1*	*Celsr1*	*Cyp7b1*	*Ednrb*
	Rasa3	*Cd200r1*	*Alox5*	*Rab3il1*	*Stxbp6*
	Tchp	*Tent5a*	*Fcgrt*	*Ifitm1*	*Tppp3*
	Crip2	*Il12a*	*Hip1*	*Rab27a*	*Kdr*
	Ddit4	*Tpcn1*	*Gramd4*	*Runx1*	*Cx3cr1*
	Snx21	*Flt3*	*Cst3*	*Bst2*	*Siglech*
PC endothelial cell	*Rspo3*	*Wnt9b*	*Wnt2*	*Kit*	*Thbd*
	Cdh13	*Fabp4*			
PP endothelial cell	*Dll4*	*Msr1*	*Efnb2*	*Ltbp4*	*Ntn4*
	Adam23				
PC hepatocyte	*Cyp2e1*	*Glul*	*Axin2*	*Ppara*	
PP hepatocyte	*Cyp2f2*	*Sds*	*Mfsd2a*	*Gls2*	*Aldh1b1*
	Pck1	*Hal*			
$CD8^+$ Tex	*Epha3*	*Gzmk*	*Ret*	*Ier3*	*Rasgef1b*
	Adam19	*Esm1*	*Pdcd1*	*Mxi1*	*Gm30211*
	Rasgef1a	*Rgs16*	*Dscam*	*Eomes*	*Rbm47*
	Zdhhc2	*Ptpn13*	*Trps1*	*Cdk6*	*Gm16226*
	Gxylt1	*Alcam*	*Gm11802*	*A430093F15Rik*	*E130308A19Rik*
	Atxn1	*Ccr5*	*Itgad*	*Runx2*	*Lag3*
	Tigit	*Tox*	*Havcr2*	*Cd8a*	*Cd8b1*
$CD4^+$ Treg	*Il1rl1*	*Foxp3*	*Slc24a3*	*Ikzf2*	*Itgb8*
	Lrrc32	*Areg*	*Gm13684*	*Itgav*	*Ctla4*
	Tnfrsf4	*Ttn*	*Lamc1*	*Neb*	*Sh3rf1*
	Dusp4	*Mctp1*	*Il1r2*	*Ikzf4*	*Dst*
	Cep112	*Hdac9*	*Nrp1*	*Rin2*	*Ankrd6*
	Cd83	*Plcl1*	*Glrx*	*Gm29114*	*Il2ra*
	Cd4				
Naive B	*Cables1*	*Egr1*	*Gm16341*	*Tmem108*	*Egr3*
	Satb1	*Dmxl1*	*Prim2*	*Dipk1a*	*Fndc9*
	B3gnt7	*Cfap43*	*Fyn*	*Slc16a6*	*Nr4a1*
	Il21r	*Retreg1*	*Maml3*	*Nr4a3*	*Ddx3y*
	Irs2	*Heg1*	*Vps37b*	*Neurl3*	*Uty*
	Scd1	*Maf*	*Ighd*	*Cd19*	*Cd79a*
	Ms4a1				
Ngp⁺ Neu	*Camp*	*Ngp*	*Ltf*	*Chil3*	*Adpgk*
	Cd177	*Itgb2l*	*Cdadc1*	*Abca13*	*Acvrl1*
	Dach1	*Ckap4*	*Cybb*	*Ifitm6*	*Ncam1*
	Trip4	*1700047M11Rik*	*Mgst2*	*Stxbp5*	*Mcu*
	Flot2	*Serpinb1a*	*Plscr1*	*Atxn10*	*Slc25a24*

	Fosl1	*Mmp8*	*Mmp25*	*Lcn2*	*Retnlg*
NK	*Irf8*	*Klra8*	*Klra4*	*Adamts14*	*Klrb1b*
	Gzma	*Klri2*	*Itga2*	*Eya1*	*Tyrobp*
	Ncr1	*Vegfa*	*Itgam*	*Ccl3*	*Prf1*
	Fcer1g	*Klrb1c*	*Cdk14*	*Lrmda*	*Dhrs3*
	Spry2	*Serpinb9b*	*Klre1*	*Klf12*	*Angpt1*
	Cma1	*Car2*	*Aoah*	*Fam184a*	*Gab2*
Spp1^{+} Mac	*Spp1*	*Gpnmb*	*Fabp5*	*Cd63*	*Gdf15*
	Bcar3	*Cstb*	*Fabp4*	*4930430E12Rik*	*Il7r*
	Anxa4	*Plxna1*	*Arhgap10*	*Emp1*	*Cxcl2*
	Acod1	*Airn*	*Ftl1*	*Lhfpl2*	*Ctsk*
	Il1rn	*St18*	*Mmp14*	*Igf2r*	*Uap1l1*
	Ctsd	*Lgals1*	*Adgre1*		
CD8^{+} Tn	*1700062C10Rik*	*Fam241a*	*Gria3*	*Auts2*	*Gm43698*
	Ccr9	*Neto2*	*Tdrp*	*Dapl1*	*Ccr7*
	Slc6a19	*Gm14085*	*Bend4*	*Pakap.1*	*Syde2*
	Actn1	*Patj*	*Rgcc*	*Lef1*	*Tmem108*
	Cmah	*Dpp4*	*Plaur*	*Usp28*	*Ifngr2*
	Cd8b1	*Rapgef4*	*Mettl8*	*Cdc14b*	*Ift80*
	Cd8a	*Sell*			
CD8^{+} Tcm	*Slc16a2*	*Yes1*	*Gzmm*	*Gm15614*	*Ms4a4c*
	Dlg5	*Fcgrt*	*Ifngas1*	*Ly6c2*	*Prss12*
	Bend4	*Gm44174*	*Cdc14b*	*Bbs9*	*Cd8b1*
	Acvr2a	*Fgf13*	*Sidt1*	*Usp18*	*1810034E14Rik*
	Hdgfl3	*Atp8b4*	*Dmrta1*	*Neto2*	*Crtam*
	Rttn	*Rasgef1b*	*2610035D17Rik*	*Jaml*	*Rnf138*
	Cd8a	*Sell*	*Cd44*		
CD8^{+} Tem	*Cx3cr1*	*Gm44174*	*Cd8a*	*Ccl5*	*Smpdl3b*
	Lamc1	*Gm44175*	*Nebl*	*Armc7*	*Cd8b1*
	Plekha6	*Klrc1*	*Klrg1*	*Lilr4b*	*Zeb2*
	Med12l	*Abcb1b*	*Lilrb4a*	*Ncald*	*Lgals3*
	Lgals1	*Rpa2*	*Rbm47*	*Slamf7*	*S100a4*
	Bcl2a1d	*Smyd1*	*S1pr5*	*Dock5*	*Ly6c2*
	Cd44				
Gluconeogenesis	*Eno2*	*G6pc2*	*Pgam1*	*Aldoc*	*Pgam2*
	Fbp2	*Tpi1*	*Slc37a1*	*Pcx*	*Pck1*
	Aldob	*Aldoa*	*Pgk2*	*Slc37a4*	*Slc37a2*
	G6pc3	*Gpi1*	*Pck2*	*Eno4*	*Gapdh*
	Eno3	*Gapdhs*	*Pgk1*	*Fbp1*	*G6pc1*
	Bcat1	*Bcat2*	*Sds*	*Sdsl*	*Gpt2*

Biosynthesis of amino acids	*Acy1*	*Asl*	*Arg1*	*Arg2*	*Ass1*
	Glul	*Gls*	*Glud1*	*Got1*	*Got2*
	Nos1	*Nos2*	*Nos3*	*Otc*	*Gls2*
	Nags	*Cps1*	*Cad*	*Gpt*	*Got1l1*
	Il4i1b	*Lao1*	*Il4i1*	*Pah*	*Tat*
	4930438A08Rik				
Insulin signaling pathway	*Acacb*	*Phkb*	*Gck*	*Rhoq*	*Ppp1r3e*
	Prkaa1	*Trip10*	*Acaca*	*Rapgef1*	*Prkaa2*
	Prkab2	*Prkag2*	*Braf*	*Pygb*	*Phka2*
	Pygl	*Raf1*	*Akt1*	*Akt2*	*Araf*
	Bad	*Calm1*	*Calm2*	*Calm3*	*Cbl*
	Socs3	*Socs1*	*Crk*	*Crkl*	*Eif4e*
	Eif4ebp1	*Elk1*	*Ppp1r3a*	*Fasn*	*Fbp2*
	Fbp1	*Flot1*	*Flot2*	*G6pc1*	*G6pc2*
	Grb2	*Gys1*	*Hk1*	*Hk2*	*Hras*
	Ikbkb	*Inppl1*	*Ins1*	*Ins2*	*Insr*
	Irs1	*Irs3*	*Irs4*	*Kras*	*Lipe*
	Mknk1	*Mknk2*	*Nras*	*Pck1*	*Pde3b*
	Pdpk1	*Phka1*	*Phkg1*	*Pik3ca*	*Pik3cd*
	Pik3r1	*Pik3r2*	*Pik3r3*	*Prkaca*	*Prkacb*
	Prkci	*Prkcz*	*Pklr*	*Ppargc1a*	*Ppp1ca*
	Ppp1cb	*Ppp1cc*	*Prkab1*	*Prkag1*	*Prkar1a*
	Prkar1b	*Prkar2a*	*Prkar2b*	*Ptpn1*	*Ptprf*
	Pygm	*Rheb*	*Rps6*	*Sorbs1*	*Shc1*
	Shc3	*Slc2a4*	*Sos1*	*Sos2*	*Srebf1*
	Cblb	*Hk3*	*Inpp5a*	*Hkdc1*	*Gm4796*
	Shc2	*Socs2*	*Eif4e1b*	*Tsc2*	*Ppp1r3d*
	Gys2	*Akt3*	*Sh2b2*	*Prkag3*	*Ppp1r3b*
	Map2k1	*Map2k2*	*Mapk1*	*Mapk10*	*Mapk3*
	Mapk8	*Mapk9*	*Eif4e2*	*Shc4*	*Irs2*
	Ppp1ccb	*Calm5*	*Ppp1r3c*	*Exoc7*	*Ppp1r3f*
	Foxo1	*Gsk3b*	*Mtor*	*Rps6kb2*	*Tsc1*
	Socs4	*G6pc3*	*Phkg2*	*Calml3*	*Rps6kb1*
	Rptor	*Pck2*	*Pik3cb*	*Calml4*	*Calm4*
Oxaloacetate metabolic process	*Acly*	*Cs*	*Got1*	*Got2*	*Mdh2*
	Mdh1	*Pck1*	*Pcx*	*Nit2*	*Csl*
	Pck2	*Mdh1b*			
	Gpat4	*Pnpla3*	*Apoc3*	*C3*	*Dgat1*
	Acsl1	*Lpin1*	*Gnpat*	*Gpam*	*Gpld1*
	Gk	*Ldlr*	*Lpl*	*Sik1*	*Pck1*

Triglyceride biosynthetic process	*Rgn*	*Nr1h4*	*Scarb1*	*Srebf1*	*Tcf7l2*
	Gpat2	*Acsl6*	*Thrsp*	*Nr1h3*	*Nr1h2*
	Lpgat1	*Fitm2*	*Gpat3*	*Mogat2*	*Slc27a1*
	Agmo	*Cnep1r1*	*Acsl5*	*Acsl4*	*Lpin2*
	Lpin3	*Plin5*	*Ctdnep1*	*Agpat2*	*Dgat2*
	Mogat1	*Tmem68*	*Tmx1*	*Pck2*	*Mfsd2a*
	Kat5	*Sirt1*			
Response to glucagon	*Adcy8*	*Cdo1*	*Creb1*	*Cry1*	*Gcg*
	Gcgr	*Gnas*	*Hmga1*	*mt-Cytb*	*Prkaca*
	Prkar1a	*Cyc*			
Response to alcohol	*Ccl19-ps5*	*Ccl19-ps4*	*Ccl21b*	*Hsd3b9*	*Mir327*
	Mir92b	*Mir1224*	*Mir1198*	*Adh6b*	*Ccl21e*
	Ccl21f	*Ccl19-ps1*	*Ccl19-ps6*	*Ccl21d*	*Dynapl1*
	Adcy3	*Ces1d*	*Rps6-ps4*	*Akr1c18*	*Akr1c14*
	Prkaa1	*Acaca*	*Prkaa2*	*Glra3*	*Nr3c2*
	Aanat	*Chrna7*	*Chrnb2*	*Slc5a5*	*Adam15'*
	Adcy6	*Adcy7*	*Adcy8*	*Adh1*	*Adh7*
	Ahr	*Akt1*	*Aldh1a1*	*Aldh2*	*Akr1c20*
	Apobec1	*Arsa*	*Hnrnpd*	*Bcl2*	*Bcl2l1*
	Blm	*Brca1*	*Casp8*	*Ctnna1*	*Ctnnb1*
	Cdk1	*Cdh1*	*Cdk4*	*Cdo1*	*Ces1g*
	Cftr	*Ccr7*	*Cnr1*	*Comt*	*Creb1*
	Crhbp	*Crhr1*	*Csn1s1*	*Cyp27b1*	*Cyp7a1*
	Slc6a3	*Dbh*	*Drd2*	*Drd3*	*Efna5*
	Epha5	*Eps8*	*Ces1c*	*Ces1e*	*F7*
	Fdx1	*Fyn*	*G6pd2*	*G6pdx*	*Ggh*
	Glra1	*Gnai1*	*Gnas*	*Gnrh1*	*Got2*
	Gpld1	*Grin1*	*Grin2a*	*Grin2b*	*Gk*
	Hnrnpk	*Hoxa1*	*Hpgd*	*Hsd3b1*	*Hsd3b2*
	Hsd3b3	*Hsd3b4*	*Hsd3b5*	*Hsd3b6*	*Htr1b*
	Htr7	*Igf1*	*Igf1r*	*Il2*	*Inhba*
	Inhbb	*Itpr2*	*Jup*	*Kcnmb1*	*Klf9*
	Lipa	*Lrp6*	*Alad*	*Recql5*	*Mlc1*
	Smad2	*Nfe2l1*	*Oprm1*	*Prkca*	*Prkce*
	Prkd1	*Ccl21a*	*Ptch1*	*Pten*	*Ptgdr*
	Ptger2	*Ptger3*	*Ptger4*	*Ptgfr*	*Rad51*
	Rara	*Rps6*	*Scnn1a*	*Scnn1b*	*Scnn1g*
	Ccl7	*Sdf4*	*Sgk1*	*Shh*	*Slc12a3*
	Sod1	*Serpina1a*	*Serpina1b*	*Serpina1c*	*Serpina1d*
	Serpina1e	*Gramd1c*	*Adcy2*	*Tacr1*	*Hnf1a*

	Unc79	*Tgfb1*	*Tgfbr1*	*Tgfbr2*	*Tgfbr3*
	Th	*Tlr4*	*Tnfsf4*	*Spidr*	*Adcy5*
	Npas4	*Lct*	*Zfp212*	*P2ry6*	*Ces1f*
	Gramd1b	*Fancb*	*Glra2*	*Scn11a*	*Ccl19*
	Grin3a	*Fkrp*	*Ces1a*	*Map4k1*	*Abat*
	Smo	*Ces1b*	*Mir9-1*	*Mir18*	*Mir20a*
	Mir10b	*Mir130a*	*Mir132*	*Mir145a*	*Mir152*
	Mir154	*Mir196a-1*	*Mir199a-1*	*Mir214*	*Mir27b*
	Mir29c	*Mir30a*	*Mir30c-1*	*Mir125b-1*	*Mir200a*
	Mirlet7a-1	*Mirlet7g*	*Gramd1a*	*Stk39*	*Eef1b2*
	Akap8	*Sphk2*	*P2ry4*	*Trp53inp1*	*Ireb2*
	Ccl19-ps3	*Borcs7*	*Golph3*	*Gpr155*	*Usp46*
	Hdac8	*Akr1cl*	*Osbpl7*	*Lancl2*	*Mir199a-2*
	Mir221	*Mir222*	*Mir106a*	*Mir30e*	*Mir362*
	Mir7-2	*Mir93*	*Mir181b-1*	*Mir100*	*Mir10a*
	Mir31	*Mir339*	*Mir7-1*	*Mir181b-2*	*Mir17*
	Mir296	*Mir322*	*Mir331*	*Mir342*	*Mir351*
	Mir466	*Mir20b*	*Mir106b*	*Mir25*	*Mir34c*
	Mir125b-2	*Mir196a-2*	*Mir30c-2*	*Mirlet7a-2*	*Mir9-2*
	Mir9-3	*Mir652*	*Mir330*	*Larp1*	*Mir709*
	Grip1	*Mir496a*	*Mir690*	*Mir92-1*	*Mir615*
	Dynap	*Ces1h*	*Arpc2*	*Aacs*	*Mir744*
	Akr1c6	*Sirt1*	*Hrh3*		
Steroid hormone biosynthesis	*Cyp3a41b*	*Cyp3a59*	*Cyp2c69*	*Hsd3b8*	*Hsd3b9*
	Ugt2b38	*Ugt2b34*	*Akr1c18*	*Cyp2c50*	*Cyp11b1*
	Ugt2b37	*Hsd17b11*	*Comt*	*Cyp11a1*	*Cyp11b2*
	Cyp17a1	*Cyp19a1*	*Cyp1a1*	*Cyp1a2*	*Cyp1b1*
	Cyp21a1	*Cyp2b10*	*Cyp2b13*	*Cyp2b19*	*Cyp2b9*
	Cyp2c29	*Cyp2c37*	*Cyp2c38*	*Cyp2c39*	*Cyp2c40*
	Cyp2d10	*Cyp2d9*	*Cyp2e1*	*Cyp3a11*	*Cyp3a13*
	Cyp3a16	*Cyp7a1*	*Cyp7b1*	*Hsd17b8*	*Hsd11b1*
	Hsd11b2	*Hsd17b1*	*Hsd17b2*	*Hsd17b3*	*Hsd17b7*
	Hsd3b1	*Hsd3b2*	*Hsd3b3*	*Hsd3b4*	*Hsd3b5*
	Hsd3b6	*Dhrs11*	*Sult1e1*	*Akr1d1*	*Sts*
	Ugt1a2	*Ugt2b5*	*Cyp2d34*	*Cyp2c70*	*Cyp2c23*
	Ugt2b36	*Ugt2b35*	*Cyp2b23*	*Hsd17b6*	*Cyp3a44*
	Cyp2d12	*Ugt1a10*	*Ugt1a7c*	*Ugt1a5*	*Ugt1a9*
	Ugt1a6b	*Ugt1a1*	*Cyp2c54*	*Cyp2c68*	*Cyp3a41a*
	Sult2b1	*Cyp2d11*	*Cyp2c67*	*Ugt2a2*	*Hsd17b12*
	Cyp3a25	*Cyp2d22*	*Srd5a3*	*Ugt1a8*	*Cyp3a57*

	Cyp2d13	*Cyp2c66*	*Cyp2d40*	*Ugt2b1*	*Cyp2c55*
	Ugt2a3	*Cyp2c65*	*Cyp2d26*	*Akr1c21*	*Srd5a1*
	Tomt	*Ugt2a1*	*Srd5a2*	*Ugt1a6a*	
Fatty acid biosynthetic process	*Gstp-ps*	*Ptges3-ps*	*Acacb*	*Acly*	*Prkaa1*
	Apoc2l	*Tecr*	*Abhd3*	*Acaca*	*Prkaa2*
	Prkab2	*Prkag2*	*Rpp14*	*Pecr*	*Acadl*
	Acadvl	*Agt*	*Abcd1*	*Alox12*	*Alox12b*
	Alox15	*Alox8*	*Alox5*	*Acsm1*	*Apoa4*
	Apoc1	*Apoc2*	*Apoc3*	*Avp*	*Brca1*
	Elovl3	*Cyp7a1*	*Degs1*	*Edn1*	*Edn2*
	Fasn	*Gip*	*Gstm4*	*Gstp2*	*Gstp1*
	Hsd17b8	*Hnf4a*	*Lipc*	*Cd74*	*Il1b*
	Eif6	*Fabp5*	*Lipg*	*Anxa1*	*Lpl*
	Ltc4s	*Elovl6*	*Mif*	*Myo5a*	*Kat2b*
	Pla2g1b	*Pla2g2a*	*Pla2g2c*	*Pla2g4a*	*Plp1*
	Prkab1	*Prkag1*	*Ptgds*	*Ptgis*	*Ptgs1*
	Ptgs2	*Abcd3*	*Qki*	*Rgn*	*Acsm3*
	Scd1	*Scd2*	*Sphk1*	*Srebf1*	*Klhl25*
	Slc45a3	*Tbxas1*	*Hnf1a*	*Nr1h3*	*Nr1h2*
	Mcat	*Gstp3*	*Erlin1*	*Lpgat1*	*Fads2b*
	Trib3	*Wdtc1*	*Insig1*	*Daglb*	*Thnsl2*
	Acsm2	*Acsm4*	*Cbr4*	*Scap*	*Pla2g3*
	Aloxe3	*Mgll*	*Prkag3*	*Tecrl*	*Erlin2*
	Acsf3	*Ceacam1*	*Ceacam2*	*Decr2*	*Mapk9*
	Pla2g10	*Abcd2*	*Mecr*	*Pla2g4f*	*Acsm5*
	Pdk4	*Scd3*	*Hacd1*	*Dcaf5*	*Fads6*
	Acsbg2	*Scd4*	*Fa2h*	*Pibf1*	*Sco1*
	Avpr1a	*Elovl1'*	*Elovl2*	*Asah2*	*Hpgds*
	Abhd2	*Hsd17b12*	*Ptges3*	*Fads2*	*Mlycd*
	Abhd1	*Hacd3*	*Mlxipl*	*Acss2*	*Fads3*
	Ptges	*Sirt2*	*Apoa5*	*Prxl2b*	*Hacd4*
	Pnpla8	*Mid1ip1*	*Gstm7*	*Acss1*	*Elovl5*
	Ubr4	*Acot7*	*Ndufab1*	*Hacd2*	*Oxsm*
	Insig2	*Acsl3*	*Elovl7*	*Fads1*	*Acsbg3*
	Lias	*Elovl4*	*Sirt1*	*Acsbg1*	*Ptges2*
	Olah				
	Acacb	*Acad11*	*Ces1d*	*Cyp4f15*	*Abhd3*
	Adtrp	*Acat1*	*Acat2*	*Etfb*	*Etfa*
	Nudt19	*Acadl*	*Acadm*	*Acadvl*	*Acaa1a*
	Acads	*Acox1*	*Adipoq*	*Akt1*	*Akt2*

Fatty acid catabolic process	*Abcd1*	*Auh*	*Cnr1*	*Cpt1a*	*Cpt2*
	Crat	*Dbi*	*Eci1*	*Faah*	*Fabp1*
	Lpin1	*Hadh*	*Hsd17b10*	*Hao1*	*Hsd17b4*
	Irs1	*Lep*	*Lipe*	*Phyh*	*Acot8*
	Pck1	*Pex7*	*Ppard*	*Abcd3*	*Abcd4*
	Pex2	*Pex5*	*Scp2*	*Slc25a17*	*Ilvbl*
	Aldh1l2	*Twist1*	*Acat3*	*Obp2a*	*Sesn2*
	Hadhb	*Ces1f*	*Acaa1b*	*Eci2*	*Decr2*
	Slc27a2	*Slc27a4*	*Abcd2*	*Gcdh*	*Abcb11*
	Etfbkmt	*Acsbg2*	*Acad12*	*Irs2*	*Acsl5*
	Echdc2	*Acaa2*	*Echdc1*	*Abhd2*	*Hao2*
	Ivd	*Mlycd*	*Mtor*	*Hacl1*	*Abhd1*
	Cyp4f40	*Cyp4f14*	*Lpin2*	*Lpin3*	*Aig1*
	Etfdh	*Lonp2*	*Plin5*	*Decr1*	*Nudt7*
	Mtln	*Eci3*	*Bdh2*	*Acot7*	*Tysnd1'*
	Acad10	*Pex13*	*Crot*	*Acoxl*	*Ehhadh*
	Pck2	*Mfsd2a*	*Acox3*	*Acox2*	*Echs1*
	Hadha'				
Tyrosine metabolism	*Il4i1b*	*Lao1*	*Maob*	*Adh1*	*Adh7*
	Adh5	*Aldh3a1*	*Aoc3*	*Aox1*	*Comt*
	Dbh	*Dct*	*Ddc*	*Fah*	*Il4i1*
	Got1	*Got2*	*Gstz1*	*Hgd*	*Hpd*
	Maoa	*Mif*	*Pnmt*	*Aox2*	*Th*
	Tpo	*Tyr*	*Tyrp1*	*Tat*	*Aoc2*
	Adh4	*Aldh3b2*	*Aldh3b1*	*Fahd1*	*Aox3*
	Aox4	*Aldh3b3*	*4930438A08Rik*	*Got1l1*	*Tomt*
SASP	*Il1a*	*Il1b*	*Il6*	*Cxcl15*	*Il11*
	Il33'	*Tnfsf13b*	*Ccl2*	*Ccl5*	*Cxcl1*
	Cxcl2	*Cxcl3*	*Cxcl5*	*Cxcl10*	*Cxcl11*
	Cxcl14	*Tgfb1*	*Tgfb2*	*Tgfb3*	*Gdf15*
	Hgf	*Mmp3*	*Igfbp3*	*Lif*	*Isg15*
Replicative senescence	*Atm*	*Atr*	*Cdkn1a*	*Cdkn2a*	*Chek1*
	Chek2	*Ctc1*	*Ercc1*	*Mme*	*Pla2r1*
	Romo1	*Serpine1*	*Tert*	*Trp53*	*Wnt16*
	Wrn				
	Abi3	*Abl1*	*Akt3*	*Arg2*	*Atm*
	B2m	*Bcl2l12*	*Bcl6*	*Arntl*	*Bmpr1a*
	Brca2	*Calr*	*Cdk2*	*Cdk6*	*Cdkn1a*
	Cdkn1b	*Cdkn2a*	*Cdkn2b*	*Cgas*	*Cited2*
	Comp	*Dnaja3*	*Ecrg4*	*Eef1e1*	*Fbxo4*

Cellular senescence	*Fbxo5*	*Fzr1*	*Gins3*	*H2-M3*	*H2-Q10*
	H2-Q7	*Hmga1*	*Hmga2*	*Hras*	*Id2*
	Ing2	*Kat5*	*Kat6a*	*Kir3dl2*	*Kras*
	Lmna	*Magea14*	*Map2k1*	*Map2k3*	*Map2k4*
	Map2k6	*Map2k7*	*Map3k5*	*Mapk10*	*Mapk11*
	Mapk14	*Mapk8*	*Mapk9*	*Mapkapk5*	*Mif*
	Mnt	*Morc3*	*Ndufs6*	*Nek4*	*Nek6*
	Npm1	*Nsmce2*	*Nuak1*	*Nup62*	*Opa1*
	Pawr	*Pla2r1*	*Plk2*	*Pml*	*Pnpt1*
	Prelp	*Prkcd*	*Prmt6*	*Pten*	*Rbl1*
	Rsl1d1	*Sirt1*	*Sirt6*	*Smc5*	*Smc6*
	Spi1	*Srf*	*Suv39h1*	*Tbx2*	*Tbx3*
	Terf2	*Tert*	*Top2b*	*Trp53*	*Trp63*
	Twist1	*Ulk3*	*Vash1*	*Wnt1*	*Wnt16*
	Wrn	*Ybx1*	*Ypel3*	*Zkscan3*	*Zmiz1*
	Zmpste24	*Zfp277*			

Supplemental Table 2. Upregulation of triglyceride anabolism-related genes in old PP hepatocytes

ST data old PV region vs young PV region					
Gene	p_val	avg_log2FC	pct.1	pct.2	p_val_adj
Mfsd2a	0.00E+00	3.5423941	0.967	0.173	0.00E+00
Lpin1	0.00E+00	2.0420249	0.959	0.656	0.00E+00
Thrsp	9.61E-269	0.8767147	0.988	0.979	3.40E-264
Pnpla3	1.10E-52	0.6502875	0.117	0	3.89E-48
Pck1	6.99E-296	0.5618083	0.997	0.994	2.47E-291
Srebf1	1.94E-37	0.4061646	0.941	0.93	6.88E-33

ST data old PV region vs old CV region					
Gene	p_val	avg_log2FC	pct.1	pct.2	p_val_adj
Pck1	0.00E+00	0.6611318	0.997	0.995	0.00E+00
Lpin1	1.22E-63	0.4963916	0.959	0.915	4.15E-59
Thrsp	3.02E-128	0.4561042	0.988	0.986	1.03E-123
Mfsd2a	8.44E-56	0.3982096	0.967	0.942	2.88E-51
Pnpla3	4.82E-17	0.3628386	0.117	0.046	1.64E-12
Srebf1	3.05E-29	0.2880631	0.941	0.907	1.04E-24

snRNA-seq old PP hepatocyte vs old PC hepatocyte					
Gene	p_val	avg_log2FC	pct.1	pct.2	p_val_adj
Lpin1	0.00E+00	0.8155219	0.683	0.624	0.00E+00
Pnpla3	2.97E-95	0.7641802	0.188	0.145	7.43E-91
Mfsd2a	5.535526e-318	0.7505703	0.461	0.38	1.386151e-313
Thrsp	1.95E-254	0.7411188	0.41	0.342	4.89E-250
Srebf1	9.31E-181	0.4929365	0.29	0.283	2.33E-176
Pck1	0.00E+00	0.339179	0.992	0.982	0.00E+00
